# Biomaterial‐Based Emergency Intervention for Secondary Spinal Cord Injury

**DOI:** 10.1002/smsc.202500125

**Published:** 2025-08-27

**Authors:** Jincheng Li, Qingzheng Zhang, Zongtai Liu, Weiguo Xu, Changfeng Fu, Jianxun Ding

**Affiliations:** ^1^ Department of Spine Surgery, Center of Orthopedics The First Hospital of Jilin University 1 Xinmin Street Changchun 130061 P. R. China; ^2^ State Key Laboratory of Polymer Science and Technology Changchun Institute of Applied Chemistry Chinese Academy of Sciences 5625 Renmin Street Changchun 130022 P. R. China; ^3^ Key Laboratory of Polymer Ecomaterials Changchun Institute of Applied Chemistry Chinese Academy of Sciences 5625 Renmin Street Changchun 130022 P. R. China; ^4^ School of Applied Chemistry and Engineering University of Science and Technology of China 96 Jinzhai Road Hefei 230026 P. R. China

**Keywords:** biomaterial, clinical application, emergency intervention, pathological change, spinal cord injury therapy

## Abstract

Timely therapeutic interventions for acute spinal cord injury (SCI) are critical for enhancing long‐term neurological and functional outcomes by limiting injury progression. However, current clinical strategies, such as methylprednisolone (MS) shock therapy and spinal decompression surgery, often yield suboptimal results. Moreover, MS administration is linked to severe systemic side effects, including pneumonia and gastrointestinal bleeding, while determining the optimal timing for decompression surgery remains challenging. Therefore, developing innovative therapeutic approaches is essential. Biomaterials, with their advanced drug delivery capabilities and unique biochemical properties, modulate cell behaviors and regulate the microenvironments of injured spinal cord, offering a promising treatment avenue for acute SCI. This review highlights the dynamic changes in SCI tissue during the initial phases and examines cutting‐edge biomaterial‐based emergency interventions, including the limitation of inflammation, reduction of excitotoxicity, restoration of the blood−spinal cord barrier, and inhibition of scar formation. Additionally, it addresses the challenges and opportunities of translating these innovations from basic research to clinical practice, thereby guiding future developments in clinically viable biomaterials.

## Graphical abstract

Emergency intervention using advanced biomaterials targets key pathological mechanisms to block secondary spinal cord injury (SCI) by inhibiting inflammation, reducing excitotoxicity, restoring the blood−spinal cord barrier, and limiting scar formation, which provides significant neural protection and promotes neural regeneration, offering promising strategies for the treatment of acute SCI and valuable insights for future clinical translation.

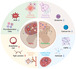

## 1 Introduction

Spinal cord injury (SCI) is a major public health issue associated with high disability and mortality rates. The number of cases continues to rise, with an estimated 27 million people affected globally and over 935 000 new cases reported annually. Traffic accidents and falls are the leading causes of SCI.^[^ ^1^, ^2^, ^3^ ^]^ SCI typically results in a time‐dependent loss of neurological function, highlighting the critical need for timely intervention, which directly influences patient survival and recovery outcomes.^[^ ^4^ ^]^ Clinical data indicate that patients receiving prehospital external fixation and transport to specialized SCI units within 24 h have a significantly lower risk of secondary complications compared to those experiencing delays. Delayed treatment increases the risk of complications by 2.5‐fold.^[^ ^5^ ^]^

Current treatments for acute SCI include high‐dose methylprednisolone (MS) shock therapy and early surgical decompression.^[^ ^6^ ^]^ MS exerts its effects by reducing spinal cord inflammation through inhibition of lipid peroxidation, pro‐inflammatory cytokines, and immune cell activity. However, high‐dose MS is associated with complications, such as infections and hyperglycemia, and lacks evidence that it offers long‐term neurological benefits for SCI patients.^[^ ^7^ ^]^ Early decompression surgery aims to relieve mechanical pressure and minimize secondary injury cascades.^[^ ^6^ ^]^ However, more than 50% of patients cannot receive surgery within the critical 24‐h window, and the long‐term effects of decompression on functional recovery remain uncertain.^[^ ^8^, ^9^ ^]^ As a result, there is an urgent need for more effective and reliable treatment strategies.

Biomaterials, due to their unique physicochemical properties, offer innovative opportunities for SCI treatment.^[^ ^10^, ^11^ ^]^ For example, stimuli‐responsive polymers modify anti‐inflammatory drugs, enabling precise spatiotemporal drug release.^[^ ^12^, ^13^ ^]^ Developing nanoformulations further optimizes therapeutic delivery by improving administration routes, enhancing efficacy, and minimizing side effects.^[^ ^14^, ^15^ ^]^ Notably, nanomedicines potentially cross the blood−spinal cord barrier (BSCB) and cell membranes.^[^ ^16^ ^]^ Additionally, multifunctional hydrogels, with superior mechanical flexibility, low immunogenicity, and biocompatibility, serve as ideal scaffolds to replace the damaged extracellular matrices (ECMs) and promote tissue regeneration after SCI.^[^ ^17^, ^18^ ^]^ Some biomaterials also possess unique chemical and biological advantages, such as the reactive oxygen species (ROS)‐scavenging ability of nanozymes and the damage‐associated molecular pattern (DAMP)‐adsorption capability of positively charged polymers.^[^ ^19^, ^20^ ^]^ Despite these advancements, the effectiveness of biomaterials remains limited due to the complexity of SCI pathology and an incomplete understanding of the injury mechanisms.

**Scheme 1:**
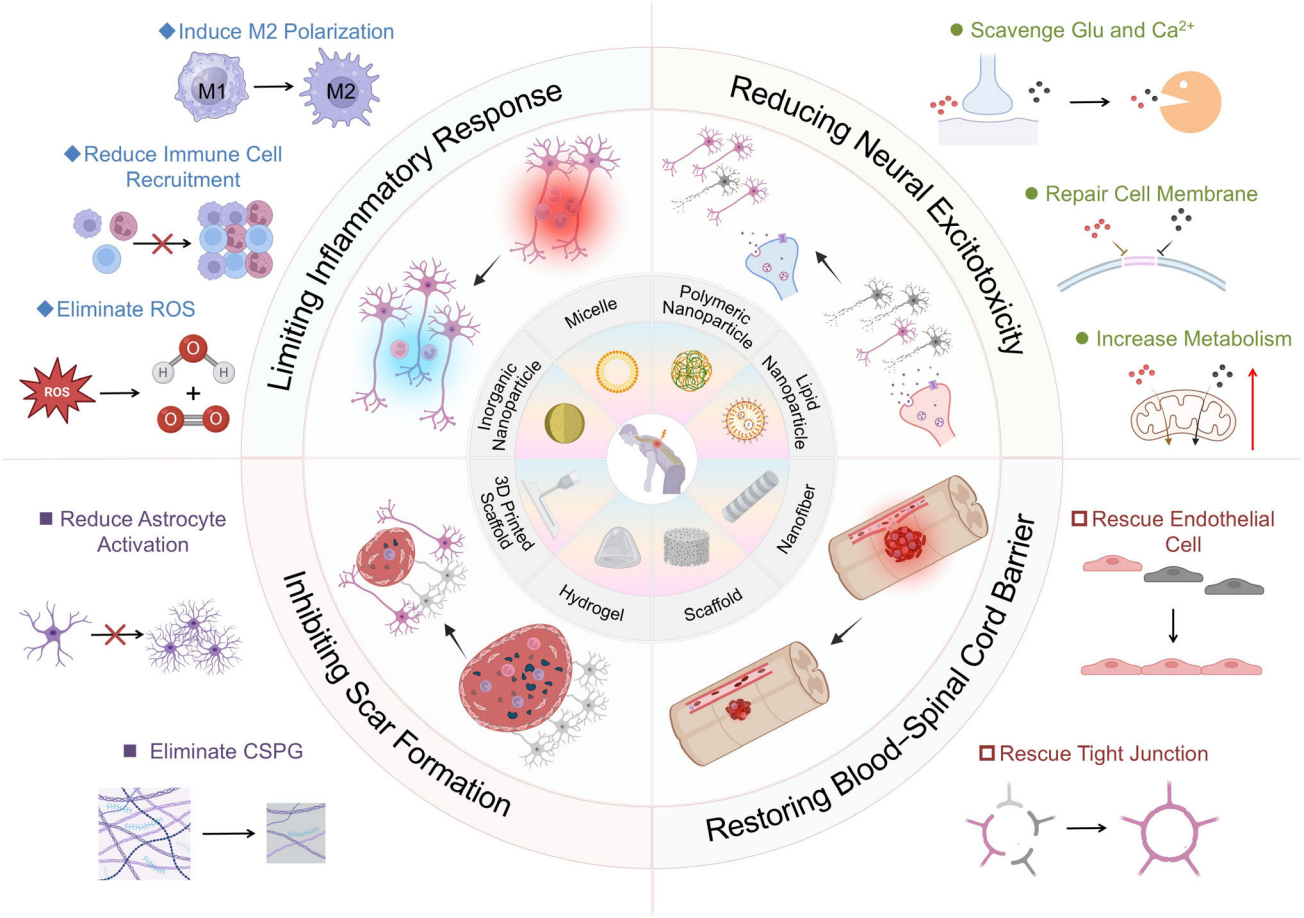
Biomaterials were used in emergency interventions of acute SCI to achieve four goals, including limiting inflammatory responses, reducing neural excitotoxicity, restoring the BSCB, and inhibiting scar formation. Created inBioRender. J. Li (2025) BioRender. com/bj2815qrut.

This review first outlines the dynamic changes in cellular and ECMs following SCI. The latest advances in biomaterials designed for use in early intervention were then systematically evaluated, with the following objectives: mitigating excessive immune responses, reducing excitotoxicity, restoring BSCB integrity, and inhibiting scar formation (**Scheme** **1**). By incorporating adaptive and compensatory strategies, a theoretical foundation was provided to guide the clinical application of biomaterials in emergency SCI intervention, ultimately promoting neuroplasticity and preserving residual functions.

## 2 Early Pathological Events of Spinal Cord Injury and their Subsequent Effects

**Scheme 2:**
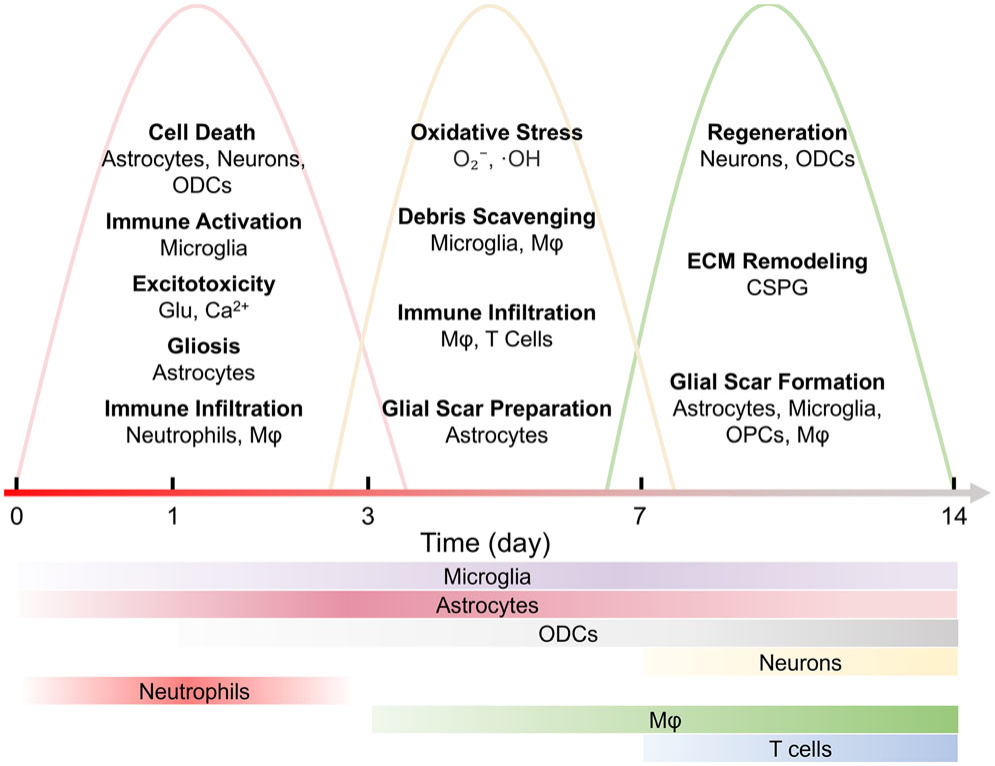
Dynamic pathological changes and cellular accumulation within 14 days.^[^ 22, 50 ^]^ The depth of the color reflects changes in the quantity of cells.

The primary injury in SCI, triggered by external mechanical forces, leads to tissue rupture, BSCB disruption, hemorrhaging, neuronal and glial cell death, and the release of cellular debris.^[^ ^6^ ^]^ The subsequent secondary injury, which arises from the primary insult, involves edema, oxidative stress (e.g., O_2_^−^, OH, and NO), excitotoxicity, ionic imbalance, cytokine storms, and glial scar formation. Secondary injury persists for months, constituting a major barrier to neuronal survival and regeneration.^[^ ^21^ ^]^ During the acute phase, significant spatial and temporal changes occur, affecting the composition and structure of spinal cord cells, ECMs, and BSCB, thereby propagating secondary damage. Most central nervous system (CNS) cell populations undergo marked depletion, with damage primarily concentrated at the injury epicenter within one day post‐injury (1 dpi).^[^ ^22^ ^]^ Understanding these dynamic mechanisms is crucial for developing effective early therapeutic strategies. This section delineates intercellular interactions, acute‐phase cell subtype transformations, and ECM/BSCB disruption‐remodeling processes that underpin early repair (**Scheme** **2**).

### 2.1 Neural and Glial Cells

The most severe SCI damage occurs in neural and glial cells, constituting spinal cord functional parenchyma: astrocytes (35%), neurons (25%), and oligodendrocytes (ODCs, 2%), all essential for neural transmission.^[^ ^22^ ^]^

#### 2.1.1 Astrocytes Participate in Glial Scar Formation and Neuroinflammation

Newly generated astrocytes quickly replenish damaged astrocytes via two primary sources: the proliferation of surviving astrocytes (47%) and the differentiation of ependymal cells (EpCs, 53%).^[^ ^23^, ^24^ ^]^ Surviving astrocytes detect DAMPs, cytokines, and chemokines, and transition from quiescent astrocytes (PAs) to reactive astrocytes (RAs) within 3 dpi, further differentiating into scar‐forming astrocytes (SAs) by 14 dpi.^[^ ^25^, ^26^, ^27^ ^]^ PAs undergo morphological changes, such as hypertrophy and antenna extension, to become RAs (<3 dpi). RAs migrate to the injury epicenter, expressing cell adhesion molecules (e.g., N‐cadherin), and with microglial support, differentiate into SAs between 3 and 14 dpi.^[^ ^25^ ^]^ SAs localize primarily at the outer edge of glial scar, expressing high levels of glial fibrillary acidic protein (GFAP) and secreting chondroitin sulfate proteoglycans (CSPGs)—key glial scar matrix components.^[^ ^24^ ^]^ This transition depends critically on the STAT3 pathway.^[^ ^28^ ^]^ EpC‐derived astrocytes constitute 53% of newly formed astrocytes.^[^ ^23^, ^24^ ^]^ Even minor injury activates EpCs, triggering their transition from quiescence to an activated state, involving self‐replication, migrating toward the injury site, and differentiating into astrocytes. In contrast to self‐proliferating resident astrocytes, EpC‐derived astrocytes show low GFAP expression and localize predominantly within the lesion core.^[^ ^23^, ^24^ ^]^

Astrocytes play a crucial role in the inflammatory response. Activated astrocytes polarize into neurotoxic (A1) or neuroprotective (A2) subtypes, defined by complement component C3 (C3) expression, a key determinant of their inflammatory functions.^[^ ^29^ ^]^ C3^+^ A1 astrocytes, induced by microglia‐derived IL‐1α, TNFα, and C1q, outnumber C3^−^ A2 astrocytes.^[^ ^29^ ^]^ The latter promotes neuronal survival and regeneration via neurotrophic factor secretion,^[^ ^29^ ^]^ whereas A1 astrocytes exacerbate post‐SCI secondary neuronal damage.^[^ ^30^, ^31^ ^]^ Therefore, modulating astrocyte polarization represents a promising early intervention strategy.

#### 2.1.2 Neurons Induce Neural Excitotoxicity

Beyond necrotic neurons, neighboring neurons undergo programmed cell death. Neuronal apoptosis detected via DFF40/CAD immunoreactivity emerges as early as 1 h post‐injury (hpi).^[^ ^32^, ^33^ ^]^ The NeuN^+^ neuronal population declines by 21% within 2 hpi, peaking at 8 hpi.^[^ ^34^ ^]^ Neuronal death extends up to 800 μm from the epicenter.^[^ ^35^ ^]^ Impaired neurons lose homeostatic capacity, releasing excessive neurotransmitters—primarily glutamate (Glu)—which induces excitotoxicity in adjacent neurons.^[^ ^36^ ^]^ Overexcited neurons propagate calcium ion (Ca^2+^) waves, triggering ROS accumulation. ROS damage cell membranes and organelles, causing secondary neuronal death and progressive degeneration.^[^ ^36^ ^]^ Limited axonal sprouting occurs within 6 hpi, potentially mediated by calpain activation.^[^ ^37^ ^]^ Specific subtypes (e.g., Vsx2^+^ and Nfib^+^ neurons) upregulate circuit‐reorganization genes for > one month, indicating regenerative potential.^[^ ^38^ ^]^ However, endogenous neural regeneration fails due to inhibitory microenvironmental factors, including pro‐inflammatory cytokines, DAMPs, myelin‐associated inhibitors, and CSPGs.^[^ ^17^, ^39^ ^]^

#### 2.1.3 Oligodendrocytes and Oligodendrocyte Progenitor Cells Inhibit Neural Regeneration

ODCs are highly vulnerable to excitotoxicity and ROS, resulting in demyelination and myelin debris release post‐SCI.^[^ ^17^ ^]^ ODC apoptosis initiates at 1 dpi, peaking at 8 dpi.^[^ ^40^ ^]^ Myelin debris components, including myelin‐associated glycoprotein (MAG), ODC myelin glycoprotein (OMgp), and Nogo‐A, strongly inhibit neural stem cell (NSC) differentiation, neurite outgrowth, and axonal regeneration. MAG and OMgp act as autoantigens that enter the circulatory system, triggering peripheral adaptive immunity.^[^ ^41^, ^42^ ^]^ Lost ODCs are replenished mainly through OPC differentiation (97%), with minor contributions from EpCs (3%).^[^ ^24^ ^]^ Beyond differentiation, reactive oligodendrocyte progenitor cells (OPCs) participate in glial scar formation.^[^ ^43^, ^44^ ^]^ They proliferate rapidly and accumulate around the lesion, becoming the dominant cellular component of glial scars.^[^ ^43^, ^44^ ^]^ When regenerating axons approach the glial scar, reactive OPCs ensheathe them, impeding further extension.^[^ ^45^ ^]^ Additionally, elevated NG2 expression in OPCs further suppresses axonal regeneration.^[^ ^46^ ^]^

### 2.2 Immune Cells

In addition to parenchymal cells, immune cells—including resident microglia and blood‐derived macrophages (Mφ), neutrophils, and lymphocytes—play pivotal roles in acute SCI.^[^ ^47^ ^]^ While they facilitate debris clearance and wound repair, they concurrently exacerbate parenchymal damage and BSCB disruption via induced cytokine storms.

#### 2.2.1 Microglia/Macrophages Phagocytize Debris and Trigger Neuroinflammation

Microglia initiate phagocytosis and recruit immune cells during early SCI, subsequently guiding glial scar formation.^[^ ^26^ ^]^ DAMPs**—**such as nucleic acids, high‐mobility group box‐1 (HMGB1), adenosine triphosphate (ATP), and matrix debris**—**activate Toll‐like receptors on microglia, transitioning quiescent microglia to an activated state.^[^ ^48^ ^]^ Activated microglia release cytokines and chemokines that recruit circulating immune cells (including Mφ and neutrophils). Mφ**—**key phagocytes**—**migrates from the compromised BSCB to the lesion core.^[^ ^49^ ^]^ The first Mφ wave occurs around 3 dpi, peaking at 7 dpi, followed by a second wave at 14 dpi.^[^ ^50^ ^]^ In lesions, distinguishing microglia from Mφ is difficult, as both exhibit two distinct phenotypes: pro‐inflammatory M1, characterized by inducible nitric oxide synthase (iNOS) activity, and anti‐inflammatory M2, characterized by arginase activity.^[^ ^49^ ^]^ M1‐polarized cells dominate early, producing high levels of pro‐inflammatory cytokines, such as IL‐1, TNF‐α, and IL‐6, and ROS. By contrast, M2 polarization is transient, peaking around 7 dpi before returning to preinjury levels. M2‐polarized cells secrete arginase‐1, IL‐10, CD206, and TGF‐*β* to promote tissue repair, yet paradoxically hinder recovery when excessive by inducing fibrotic scars.^[^ ^49^ ^]^ Thus, a tightly regulated M1/M2 balance is critical for optimal recovery.

#### 2.2.2 Neutrophils Exacerbate Tissue Damage

Neutrophils are the first blood‐derived immune cells infiltrating the lesion core, appearing within 2 hpi, peaking at 1 dpi, and subsiding by 3 dpi in SCI models.^[^ ^51^ ^]^ Neutrophils guide Mφ migration and may directly phagocytose debris.^[^ ^52^ ^]^ However, they also exacerbate neuronal damage and BSCB disruption through degranulation and the release of neutrophil extracellular traps.^[^ ^53^, ^54^ ^]^ Neutrophil infiltration correlates with ECM deterioration within the first 3 dpi. Nevertheless, their precise neuron‐interaction mechanisms remain mechanistically elusive.

#### 2.2.3 Lymphocytes Exacerbate Neuroinflammation

Lymphocytes contribute minimally during acute SCI (unlike microglia/Mφ/neutrophils) but significantly amplify damage later (≈7 dpi) despite low abundance.^[^ ^32^ ^]^ In humans, lymphocytes are predominantly CD8^+^ and CD4^+^ T cells.^[^ ^55^ ^]^ CD8^+^ T cells release perforin, which disrupts the BSCB integrity and facilitates inflammatory infiltration. They may also release granzyme, which activates caspase‐3‐mediated neuronal apoptosis.^[^ ^56^ ^]^ CD4^+^ T cells differentiate into several subtypes, including Th1, Th2, Th17, Treg, and γδT cells.^[^ ^57^ ^]^ Pro‐inflammatory subsets (Th1/Th17) drive microglial/Mφ M1 polarization, amplifying inflammation, neuronal damage, and demyelination.^[^ ^58^, ^59^, ^60^ ^]^ Conversely, Th2 and Treg cells mitigate inflammation by secreting anti‐inflammatory cytokines, such as IL‐4 and IL‐10.^[^ ^61^, ^62^ ^]^ Th2 cells promote axonal sprouting by inducing neurotrophin‐3 (NT‐3),^[^ ^63^ ^]^ while Treg cells suppress hyperimmunity through PD‐1/CTLA‐4 expression.^[^ ^64^, ^65^ ^]^ Despite these protective subsets, the overall lymphocyte response is dominated by pro‐inflammatory activity. As lymphocyte infiltration initiates at 3 dpi, early intervention strategies targeting lymphocytes may improve outcomes by attenuating detrimental effects.

### 2.3 Extracellular Matrix and Blood−Spinal Cord Barrier

#### 2.3.1 Extracellular Matrix Remodulation Leads to Wound Healing

The ECM comprises hyaluronic acid (HA), sulfated proteoglycans, collagen, fibronectin, laminin, and tenascin‐R, providing a scaffold for neuronal progenitor proliferation, axonal guidance, and synaptic plasticity.^[^ ^66^ ^]^ During SCI, ECM disorganization exacerbates inflammation.^[^ ^67^ ^]^ Infiltrating immune cells secrete matrix metalloproteinases (MMPs), promoting ECM degradation.^[^ ^68^ ^]^

CSPGs (e.g., Brevican, Versican, NG2, Neurocan, and Phosphacan), secreted by astrocytes and OPCs, constitute major glial scar components.^[^ ^69^ ^]^ The deposition of CSPG forms glial scars, facilitating wound healing. Under normal conditions, CSPGs are expressed at low levels, but their expression increases significantly during pathological events.^[^ ^26^ ^]^ CSPGs form the primary inhibitory component of the glial scar, creating an unfavorable microenvironment for axonal regrowth. They inhibit axonal extension by interacting with receptors like protein tyrosine phosphatase sigma (PTPσ), leukocyte common antigen‐related (LAR), and Nogo66 on neuronal membranes.^[^ ^69^, ^70^ ^]^ Notably, some CSPGs (CSPG4/5) may support regeneration via chondroitin sulfate chains.^[^ ^71^ ^]^ Thus, CSPG modulation represents a therapeutic target, though mechanistic insights remain limited.

#### 2.3.2 Blood−Spinal Cord Barrier Damage Leads to Inflammatory Infiltration

The BSCB consists of endothelial cells, pericytes, basement membranes, and astrocytic foot processes, all interconnected via tight junctions (TJs) to maintain spinal cord immune isolation.^[^ ^72^, ^73^ ^]^ The BSCB compromise permits peripheral leukocytes infiltration and MMP secretion, which amplifies inflammation by degrading the ECMs.^[^ ^68^ ^]^ Post‐SCI, microvascular structures are rapidly disrupted, and the BSCB is disrupted within 5−15 min, triggering an ischemic cascade and sustained neurodegeneration.^[^ ^72^ ^]^ The BSCB compromise often results in hemorrhages, exposing spinal tissue to inflammatory cells, cytokines, and vasoactive peptides, leading to edema, cytokine storms, and cell necrosis.^[^ ^6^ ^]^ TJ integrity is crucial for controlling BSCB permeability and depends on membrane proteins, like occludins, claudins, zonula occludens (ZO) proteins, and junctional adhesion molecules.^[^ ^74^ ^]^ Thus, preserving or restoring the BSCB function is crucial to mitigate secondary injury progression.

## 3 Targets of Spinal Cord Injury Emergency Intervention Utilizing Biomaterials

SCI triggers pathophysiological events, including necrotic cell death, BSCB dysfunction, and tissue damage.^[^ ^32^ ^]^ These events lead to the release of Glu, Ca^2+^, and DAMPs, such as HMGB1, ATP, and DNA.^[^ ^6^, ^75^ ^]^ The excessive influx of Ca^2+^ impairs astrocytic Glu uptake, resulting in elevated Glu levels, which promote excitotoxicity and further cell death, perpetuating secondary injury. Concurrently, the accumulation of DAMPs activates microglia and recruits circulating immune cells, thereby contributing to the inflammatory process.^[^ ^75^ ^]^ The predominance of pro‐inflammatory (M1) Mφ and microglia, along with an imbalance between M1 and M2, exacerbates the inflammatory response and creates a hostile microenvironment that hinders recovery.^[^ ^76^ ^]^ Breakdown of the BSCB, often driven by hypoxia at the injury site, amplifies this inflammatory cascade, further releasing pro‐inflammatory factors and ECM debris.^[^ ^77^ ^]^ These mediators stimulate CSPG production, producing dense CSPG accumulation and glial scar formation by activated astrocytes. While the glial scar helps contain the injury, it also presents a significant barrier to functional recovery and tissue repair.^[^ ^26^ ^]^ This section explores emerging biomaterial‐based strategies targeting four critical early‐stage interventions: suppression of inflammation, mitigation of excitotoxicity, restoration of the BSCB, and remodeling of the glial scar.

### 3.1 Inhibition of Inflammatory Response

Early efforts to control post‐injury inflammatory response primarily focused on administering traditional anti‐inflammatory drugs, such as MS,^[^ ^78^ ^]^ minocycline (MN),^[^ ^79^, ^80^ ^]^ and azithromycin,^[^ ^81^ ^]^ through biomaterial vectors or scaffolds.^[^ ^75^, ^78^, ^82^, ^83^, ^84^, ^85^, ^86^, ^87^, ^88^, ^89^, ^90^, ^91^, ^92^, ^93^, ^94^, ^95^, ^96^, ^97^, ^98^ ^]^ **Table** **1** summarizes standard drug delivery methods and their corresponding therapeutic effects. However, because neuroinflammation has beneficial and harmful effects, indiscriminate suppression often fails to achieve the desired therapeutic outcomes. Advances in biomaterials science have led to strategies that target specific phases of inflammation, thereby enhancing neuroprotection. After SCI, the inflammatory process involves 1) activation of resident immune cells (microglia), 2) recruiting of circulating immune cells through the disrupted BSCB, 3) shifting immune cells toward a pro‐inflammatory phenotype, and 4) accumulating ROS. Targeting these key aspects could enable more precise regulation of the inflammatory response.^[^ ^6^, ^32^, ^99^ ^]^

**Table 1:** Biomaterial‐base strategies used to reduce inflammation response after SCI.

Materiala)	Modification/loaded	Outcome	Inflammatory Factor Level (Treatment/Saline Ratio)	BBB (rats)/BMS (mice) Score Improvement (Compare with Saline Group)	Limitation	Reference
Sialic acid–PEG‐PLGA copolymer nanoparticle (SAPP)	MN	Sialic acid targeted inflammatory vascular endothelial cells at the injured site. PEG repair damaged the membrane. MN acts as an anti‐inflammatory drug. Combinational effects of SAPP reduced the pro‐inflammatory factors and ROS.	0.46 TNF‐α 0.53 IL‐6 0.47 IL‐1β (ELISA, 1 dpi)	8.17 (BBB) (8 weeks)	Not paying attention to the long‐term inflammatory response.	[82]
Amino poly(lactic acid)‐oriented microsol electrospun fiber scaffold	Aldehyde‐modified cationic liposomes loading IL‐4 pDNA	pDNA‐induced IL‐4 secreted to the microenvironments and lead to the M1‐to‐M2 phenotype reprogramming of microglia/M*φ* and the reduction in pro‐inflammatory factors.	0.78 TNF‐α 0.80 IL‐1β (ELISA, 7 dpi)	7.61 (BBB) (8 weeks)	Fiber scaffolds degrade relatively quickly	[83]
PLGA‐PEG‐PLGA hydrogel	Baricitinib (JAK1/JAK2 inhibitor)	Baricitinib‐PLGA‐PEG‐PLGA hydrogel inhibits the polarization of M1 and secretion of pro‐inflammatory factors in SCI rats.	0.61 iNOS 0.60 TNF‐α (WB, 3 dpi)	8.21 (BBB) (4 weeks)	The relationship between the hardness of hydrogel and inflammation was not discussed.	[84]
Zein‐based spherical nanoparticle containing the peptide CAQK	MET	CAQK‐MET‐NPs exhibited high spinal cord‐targeted and MET delivery efficiency. They caused a sharp reduction in pro‐inflammatory and promotion of neuron survival.	0.39 IL‐6 0.30 TNF‐α (ELISA, 1 dpi)	12.07 (BBB) (8 weeks)	Not paying attention to the long‐term inflammatory response.	[85]
Carrier‐free thioketal‐linked methylprednisolone dimer@rutin nanoparticle (MP2‐TK@RU NP)	Methylprednisolone and rutin	MP2‐TK@RU NP possessed high drug‐loading and ROS‐responsive drug‐releasing capacity. They reduced the expression of Iba‐1, TNF‐α, and IL‐6 in SCI rats and protected the survived neurons	0.25 iba‐1 0.49 TNF‐α 0.17 IL‐6 (IF, 2 dpi)	7.62 (BBB) (8 weeks)	Lack of research on immune cell phenotype changes.	[78]
Carboxymethyl chitosan and gallic acid hydrogel (CSGA)	None	CSGA limited the infiltration of microglia/M*φ* and induced M2 phenotype reprogramming.	0.40 TNF‐α 0.79 IL‐6 0.26 IL‐1β (ELISA, 7 dpi)	6.50 (BBB) (8 weeks)	The relationship between the hardness of hydrogel and inflammation was not discussed.	[97]
LNPs	^IL−10^mRNA	LNP‐^IL−10^mRNA reduced the activation of microglia/M*φ* and enhanced tissue sparing.	0.80 TNF‐α (PCR, 2 dpi)	3.83 (BBB) (8 weeks)	Lack the research on immune cell phenotype changes.	[88]
Poly(ε‐caprolactone) nanofiber‐HA hydrogel composite (NHC)	None	NHC provided mechanical support to the contused spinal cord and promoted a shift toward a proregenerative M*φ* population, angiogenesis, and neurogenesis.	None	0 (BBB) (8 weeks)	The recovery of motor function is not significant.	[98]
PLGA microsphere‐alginate hydrogel	MN hydrochloride and PTX	MH reduced microglia reaction and neuronal death. PTX promoted neuronal regeneration.	0.28 ED1 (IF, 7 dpi)	3.92 (BBB) (4 weeks)	Lack the detection of inflammatory factors.	[89]
Dopamine‐chitosan‐citric acid hydrogel (CS‐CA‐DA)	None	CS‐CA‐DA promoted M*φ* polarization to the M2 phenotype and led to axonal regeneration.	None	3.71 (BBB) (7 weeks)	Lack the detection of inflammatory factors.	[92]
Nanofiber scaffold HA hydrogel patch	Methylprednisolone and Schwann cell‐derived exosomes	The composite patch inhibited inflammatory response by switching M1 to M2 polarization and protecting neurons from apoptosis.	0.46 iNOS (IF, 3 dpi)	4.237 (BBB) (4 weeks)	Lack the evaluation for long‐term SCI repair.	[93]
Silk fibroin hydrogels	S100A4	The in situ silk fibroin hydrogel recruited immune cells, S100A4 mediated M2 polarizatio.	0.46 IL‐1β 0.68 IL‐6 0.73 TNF‐α (PCR, 7 dpi)	7.34 (BBB) (4 weeks)	Lack the evaluation for long‐term SCI repair.	[94]
Mg/Al‐layered double hydroxide nanoparticle (LDH)	NT‐3	LDH‐NT‐3 induced M2 polarization of microglia/M*φ* as well as NSCs neural differentiation.	0.23 TNF‐α (WB, 7 dpi)	1.28 (BMS) (8 weeks)	Lack the research on immune cell phenotype changes of the injured spinal cord.	[90]
Retinoic acid‐bovine serum albumin‐ curcumin nanoparticle (RA@BSA@CUR NPs)	RA and CUR	CUR limited the infiltration of M*φ* and induced M2 phenotype switch as well as scavenged ROS. Retinoic acid induced axon regeneration and neurogenesis.	0.76 TNF‐α 0.85 IL‐6 (ELISA, 7 dpi)	3.66 (BMS) (8 weeks)	The frequency of administration is relatively high	[91]
LDH‐NT‐3 combined with ultrasound treatment.	NT‐3	The combined treatment reduced pro‐inflammatory cytokine expression and promoted neural differentiation of NSCs.	0.06 TNF‐α (IF, 7 dpi)	2.57 (BMS) (8 weeks)	Lack the research on immune cell phenotype changes.	[95]
MSN core and hUC‐MSC membrane (CM‐miR‐MSN)	miRNA‐124‐3p	The CM‐miR‐MSN delivery miRNA‐123‐3p to microglia and neurons, inducing M2 phenotype reprogramming and axon growth.	None	4.06 (BMS) (8 weeks)	Lack the detection of inflammatory factors.	[96]
Assembly of PLGA and resveratrol NPs with microglial membrane coating (PR@MM)	Resveratrol	PR@MM efficiently penetrated the BSCB and accumulated in the SCI lesion. PR‐NPs released toward the microenvironments and exhibited anti‐ROS and anti‐inflammation efficacy in SCI mice.	0.25 iba‐1 (IF, 28 dpi)	2.53 (BMS) (4 weeks)	Lack the detection of inflammation in the acute phase.	[86]
Rhodamine‐polyethyleneimine and PEG‐carbonyldiimidazole nanogel (NG)	Rolipram	NG was internalized only by microglia and astrocytes and limited the neuroinflammation.	None	0.53 (BMS) (3 weeks)	The recovery of motor function is not significant.	[87]

a) Abbreviation: BBB (Basso Beattie Bresnahan); BMS (Basso Mouse Scale); PEG (Polyethylene glycol); PLGA (poly (lactic‐co‐glycolic acid)); BSCB (Blood−Spinal Cord Barrier); CUR (Curcumin); ELISA (Enzyme‐Linked Immunosorbent Assay); LNP (Lipid Nanoparticle); HA (Hyaluronic Acid); MET (Metformin); MN (Minocycline); NSC (Neural Stem Cell); NT‐3 (Neurotrophin‐3); pDNA (Plasmid DNA); PTX (Paclitaxel); RA (Retinoic Acid); ROS (Reactive Oxygen Species); SCI (Spinal Cord Injury); WB (Western Blot); IF (Immunofluorescence Staining).

#### 3.1.1 Transforming Activated Microglia into Quiescent State

Microglia are the first immune cells to produce an immune response after SCI. The severe trauma often leads to excessive microglial activation.^[^ ^6^ ^]^ Therefore, transforming activated microglia into a quiescent state is an effective measure to suppress the inflammatory response.

**Figure 1:**
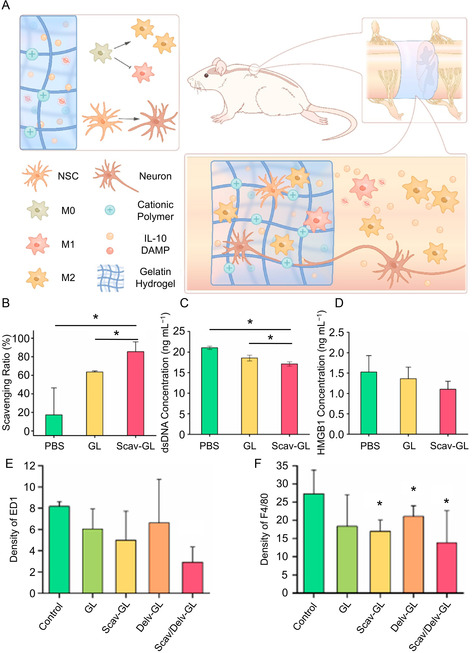
DAMPs‐scavenging scaffold reduces the activation of microglia, inducing the reduction of inflammatory response. A) DAMP‐scavenging scaffold for SCI repair. B) Total protein in DAMP solution and C) dsDNA and D) HMGB1 levels after incubation with the DAMP‐scavenging scaffold in vitro. E) ED1 and F) F4/80 expression in injured spinal cord after implantation of DAMP‐scavenging scaffold. All statistical data are represented as mean ± SD (*n* = 3; *P < 0.05, ^#^ *P* < 0.05 compared with the GL group for (E)). Reproduced with permission.^[^ ^102^ ^]^ Copyright 2022, Elsevier.

Transforming activated microglia into a quiescent state involves clearing external stimuli and modulating internal signaling pathways. DAMPs are key external stimuli that activate microglia, making DAMP clearance a crucial intervention to prevent subsequent immune responses. Cationic polymers are particularly effective at clearing negatively charged DAMPs through electrostatic interactions.^[^ ^100^, ^101^ ^]^ Shen et al.^[^ ^102^ ^]^ developed a DAMP‐scavenging scaffold by immobilizing cationic poly(amidoamine) nanoparticle (PAMAM NP) within a visible light‐cross‐linked gelatin hydrogel (**Figure** **1**A). This scaffold effectively absorbed HMGB1 and double‐stranded DNA (dsDNA) fragments, thereby reducing DAMP‐induced microglial activation and pro‐inflammatory polarization in vitro (Figure 1B,C). When implanted into injured spinal cords, the scaffold significantly reduced inflammatory cytokine production (IL‐1*β* and TNF‐α) and promoted motor function recovery in SCI mice (Figure 1D,E). The gelatin‐only scaffold notably exhibited mild DAMP‐binding activity (Figure 1B,C). Gao et al.^[^ ^103^ ^]^ further enhanced these outcomes by incorporating PAMAM NP into a hybrid hydrogel of gelatin and HA, reducing immune responses while also improving the survival and function of transplanted allogeneic spinal cord tissues.

Regulating internal signaling pathways to reverse microglial activation presents another effective strategy. Activated microglia can be suppressed by targeting inflammation‐related signaling pathways, such as the *p38 MAP kinase* and *cGAS‐STING* pathways. Stigliano et al.^[^ ^104^ ^]^ engineered a nanocomposite of a poly(lactic*‐co*‐glycolic acid) nanoparticle (PLGA NP) loaded with PF3644022, a selective MK2 inhibitor, and superparamagnetic iron oxide nanosphere for magnetic targeting and site‐specific localization. PF3644022 inhibited microglial activation by suppressing the *p38 MAP kinase* pathway, resulting in a nearly threefold reduction in the number of Iba‐1^+^ cells in vivo. A recent study identified that downregulation of mitofusin 2 (Mfn2) expression in microglia disrupts mitochondrial fusion and fission, leading to the release of mitochondrial DNA (mtDNA) after SCI.^[^ ^105^ ^]^ The mtDNA then activated microglia via the *cGAS‐STING* pathway. To address this, researchers developed mesoporous silica nanoparticle (SiO_2_ NP) coated with microglia cell membranes to deliver Mfn2 agonists, successfully reducing microglial activation and pro‐inflammatory cytokine release. Additionally, microRNAs (miRNAs) play an essential role in modulating microglial phenotypes. For instance, miRNA‐124 promotes microglial quiescence by regulating the transcription factor C/EBP*‐α* and its downstream target PU. 1. Louw et al.^[^ ^106^ ^]^ created chitosan‐based nanoparticles encapsulating miRNA‐124, resulting in a fivefold reduction in ED1^+^ cells (a marker for activated microglia/Mφ).

Additionally, targeted ablation of activated microglia has shown promising outcomes. Ma et al.^[^ ^107^ ^]^ introduced an innovative strategy using a gelatin scaffold loaded with the CSF1R inhibitor PLX3397 to deplete activated microglia at the injury site. The ablated microglia were subsequently replaced by newly formed, quiescent microglia. However, precise control of the ablation process is critical. A recent study demonstrated that excessive ablation exacerbates lesion expansion, reduces the survival of neurons and ODCs, and impairs motor function.^[^ ^108^ ^]^

#### 3.1.2 Reducing Blood‐Derived Immune Cells Infiltration

In response to injury, blood‐derived immune cells are recruited through cytokines and chemokines released by damaged tissues.^[^ ^99^ ^]^ Thus, many anti‐inflammatory therapies aim to decrease immune cell infiltration (Table 1).

Emerging research highlights immune cell reprogramming as a powerful approach to reduce immune infiltration. Studies have shown that nanoparticles with high negative charges bind to scavenger receptors on circulating immune cells, reprogramming them to migrate preferentially to the spleen rather than the site of injury. For example, Park et al.^[^ ^109^ ^]^ demonstrated that PLGA NP (500 nm diameter, zeta potential < −30 mV) was internalized by circulating monocytes and neutrophils following intravenous administration in SCI mice. The accumulation of innate immune cells at the lesion core decreased by fourfold, accompanied by a reduction in the expression of pro‐inflammatory markers and an increase in the expression of anti‐inflammatory and proregenerative genes.

**Figure 2:**
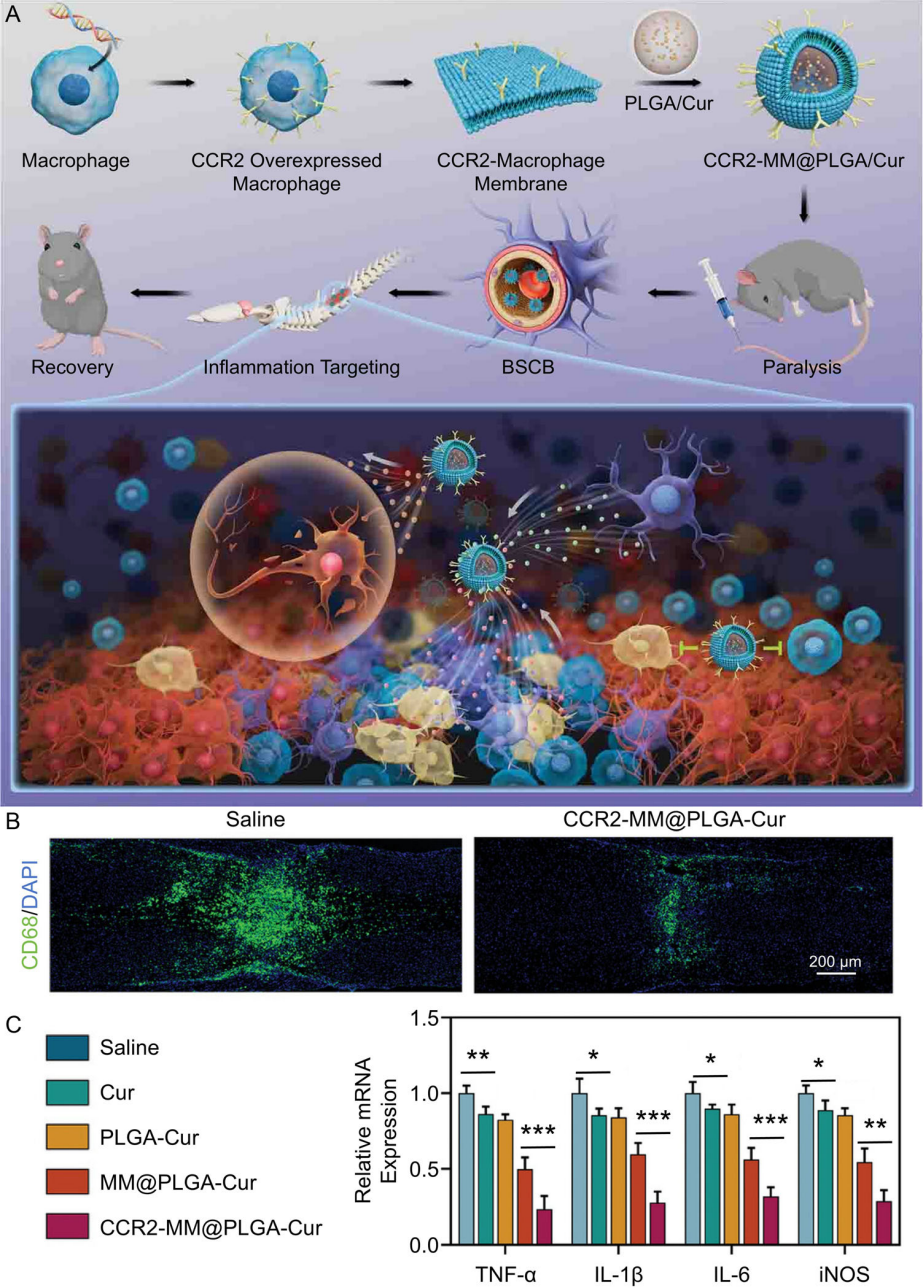
“Mφ decoy” nanoplatform reduces immune infiltration at injured site. A) “Mφ decoy” nanoplatform for SCI repair. B) Image of CD68 expression in SCI mice receiving treatments at 7 dpi. C) Relative mRNA expression levels of M1‐related genes in the spinal cord at 7 dpi. All statistical data are represented as mean ± SD (*n* = 5; *P < 0.05; **P < 0.01; ***P < 0.001). Reproduced with permission.^[^ ^110^ ^]^ Copyright 2023, Wiley‐VCH.

Nanomaterials coated with immune cell membranes mimic circulating immune cells in vivo, further limiting inflammatory infiltration at the injury sites. Gu et al.^[^ ^110^ ^]^ developed a “Mφ decoy” nanoplatform consisting of an Mφ membrane surrounding a PLGA core (**Figure** **2**A). This system reduced Mφ infiltration at the injury center by ≈60% compared to the saline‐treated group. Enhancing this strategy, the authors upregulated CCR2 expression on the Mφ membrane through genetic engineering, further decreasing Mφ infiltration by ≈75% by SEQUESTering the chemokine CCL2. Additionally, the engineered cell membrane significantly increased the nanoplatform's enrichment rate at the injury center, enhancing the anti‐inflammatory effect of the drugs carried within the PLGA core (Figure 2B,C). Similarly, Bi et al.^[^ ^111^ ^]^ designed a “neutrophil decoy” nanoplatform by coating a polydopamine nanoparticle (PDA NP) with a neutrophil membrane vesicle (NMV). The NMV neutralized various chemokines and inflammatory mediators, blocking neutrophil infiltration and activation. Following tail‐vein injection of the nanoplatform into SCI rats, neutrophil infiltration at the injury site was substantially reduced, approaching levels observed in uninjured control group.

Some biomaterials actively trigger immune cell recruitment. For instance, hydrogels rich in imidazole groups—key components of histamine—attract microglia and Mφ via interactions with histamine receptors H1R and H4R.^[^ ^112^ ^]^ Similarly, silk fibroin promotes the recruitment of neutrophils and Mφ while inducing Mφ to secrete pro‐inflammatory cytokines.^[^ ^113^, ^114^ ^]^ Such biomaterials, potentially modulating cell migration, could be engineered to mitigate immune infiltration in SCI.

#### 3.1.3 Inducing Microglia/Macrophages Polarization from M1 to M2

Since microglia and Mφ predominantly exhibit the pro‐inflammatory M1 phenotype during acute SCI, promoting their transformation to the anti‐inflammatory M2 phenotype is widely recognized as beneficial for functional recovery.^[^ ^47^, ^49^, ^76^ ^]^

Breakthrough studies have identified interferon regulatory factors (IRFs) as key modulators of Mφ phenotype switching. Among them, IRF5 is critical in promoting M1 polarization, with its high expression marking a shift toward the M1 state.^[^ ^115^ ^]^ Ma et al.^[^ ^116^ ^]^ used polyethyleneimine‐conjugated, diselenide‐bridged mesoporous silica nanoparticle (rMSN‐siRNA NP) to deliver IRF5‐targeting siRNA. Local injection of rMSN‐siRNA NP halved the number of iNOS^+^F4/80^+^ (M1 biomarker) cells and quadrupled the number of Arg1^+^F4/80^+^ (M2 biomarker) cells within the injured spinal cord. Similarly, Xiong et al.^[^ ^117^ ^]^ developed a multifunctional “pollen” nanocomposite, where IRF5‐siRNA was delivered using gelatin hydrogel loaded with “nanoflower” manganese tetroxide (Mn_3_O_4_) structure. This system not only induced M2 phenotype reprogramming but also reduced ROS accumulation and promoted neuronal regeneration across multiple functional subtypes.

**Figure 3:**
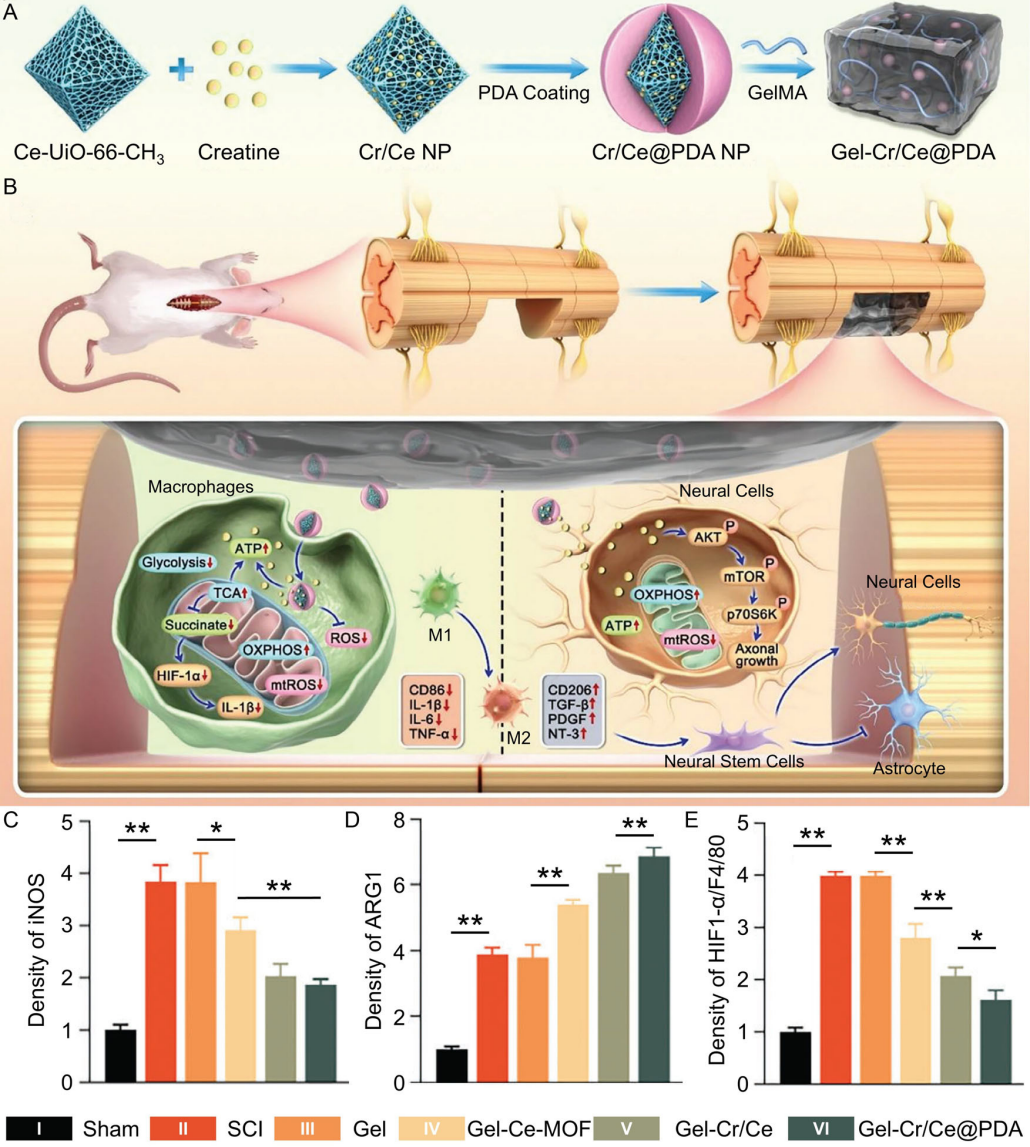
Gel‐Cr/Ce@PDA promotes M2 polarization by providing mitochondrial energy supply. A) Synthesis of Gel‐Cr/Ce@PDA. B) Gel‐Cr/Ce@PDA promotes M2 polarization by supplying mitochondrial energy. C) iNOS, D) ARG1, and E) HIF1‐α/F4/80 expression in the spinal cord after treatment with Gel, Gel‐Ce‐MOF, Cel‐Cr/Ce, and Gel‐Cr/Ce@PDA. All statistical data are represented as mean ± SD (*n* = 3; *P < 0.05, **P < 0.01). Reproduced with permission.^[^ ^119^ ^]^ Copyright 2024, Wiley‐VCH.

A novel approach to promoting M2 polarization involves replenishing energy in Mφ mitochondria. The dynamic changes in Mφ phenotypes are closely linked to mitochondrial metabolic states.^[^ ^118^ ^]^ Recently, significant attention has been directed toward reprogramming the M2 phenotype by modulating mitochondrial metabolism. Jiang et al.^[^ ^119^ ^]^ developed a system by loading creatine onto cerium‐based metal–organic framework (MOF) and encapsulating it with PDA to create a microcapsule (Cr/Ce@PDA NP). These were further embedded into GelMA hydrogel (Gel‐Cr/Ce@PDA) and implanted into the injured spinal cord (**Figure** **3**A). Creatine, an energy storage molecule, facilitates the rapid regeneration of ATP from ADP. Cr/Ce@PDA NP enhanced succinate metabolism by supplying mitochondrial energy to Mφ at the injury site. This reduction in succinate levels inhibits the *succinate/HIF‐1α/IL‐1β* signaling axis (Figure 3B), ultimately increasing the expression of M2 polarization biomarkers (Figure 3C−E). Compared to drug delivery for mitochondrial energy replenishment, the direct transplantation of M2‐derived mitochondria has shown greater efficacy. Xu et al.^[^ ^120^ ^]^ demonstrated that mitochondria (Mito) isolated from IL‐10‐induced M2 provide a more effective therapeutic strategy. To improve targeting to the injury site, they modified the mitochondria using triphenylphosphonium cations and a cysteine–alanine–glutamine–lysine peptide (Mito‐Tpp‐CAQK). Mito‐Tpp‐CAQK not only facilitated M2 phenotype reprogramming but also enhanced the phagocytosis of myelin debris. This treatment ultimately improved motor function and bladder nerve innervation in SCI mice.

#### 3.1.4 Eliminating Reactive Oxygen Species

Following SCI, ROS are produced in large quantities by activated microglia and blood‐derived immune cells. ROS include superoxide anion radicals (O^2−^), hydrogen peroxide (H_2_O_2_), hydroxyl radicals (·OH), and peroxynitrite.^[^ ^121^ ^]^ At the cellular level, excessive ROS leads to damage of mitochondrial and nuclear DNA, as well as lipid peroxidation, triggering various programmed cell death pathways.^[^ ^122^ ^]^ On a molecular level, overproduced ROS activate numerous signaling pathways, including *MAPK*, *NFκB*, and *JAK/STAT*, which exacerbate inflammation.^[^ ^123^, ^124^ ^]^ The body's ability to clear ROS relies on antioxidant enzymes, such as superoxide dismutase, catalase, and glutathione peroxidase. However, excessive ROS production overwhelms these intrinsic antioxidant mechanisms.^[^ ^125^ ^]^

The use of nanomaterials or scaffolds to deliver traditional therapeutic agents, such as curcumin (CUR),^[^ ^91^ ^]^ metformin (MET),^[^ ^126^ ^]^ rutin,^[^ ^78^ ^]^ and resveratrol,^[^ ^86^ ^]^ reduces ROS production by inhibiting the activation of immune cells in the injured spinal cord. Similarly, delivering exogenous antioxidant enzymes to enhance the tissue's ability to scavenge ROS has shown significant neuroprotective potential.^[^ ^127^, ^128^ ^]^ However, the low efficacy of traditional drugs due to nonspecific ROS targeting, along with the instability and high cost of antioxidant enzymes, limits their clinical applications. Biomaterials with unique biochemical properties, such as nanozymes and polymer‐based materials with reactive groups that scavenge ROS, offer distinct advantages for ROS clearance.

Nanozymes, a class of nanomaterials with biocatalytic functions, combine the inherent physical and chemical properties of nanomaterials with enzyme‐like catalytic activity. They are more stable and easier to produce compared to natural enzymes.^[^ ^20^ ^]^ Nanozymes exhibiting peroxidase, catalase, and superoxide dismutase activities have demonstrated excellent ROS‐scavenging capabilities and have been effectively applied in recent SCI treatments.

The most widely studied and utilized nanozymes for SCI treatment are cerium oxide nanoparticles (CONPs) and manganese oxide nanoparticles (MnO_2_ NPs). CONPs owe their oxidative catalytic abilities to variable oxidation states (Ce^3+^/Ce^4+^). Kim et al.^[^ ^129^ ^]^ demonstrated that CONPs protected neurons from oxidative damage induced by H_2_O_2_ in vitro. When different concentrations of CONPs were injected into injured spinal cords, a reduction in ED1^+^ Mφ infiltration and inflammatory markers, such as iNOS, was observed. The optimal therapeutic concentration was identified as 500–1000 μg mL^−1^. To enhance tissue repair, Zheng et al.^[^ ^130^ ^]^ embedded CONPs within poly(ε‐caprolactone) porous nanoscaffolds and transplanted them into the injured spinal cord. The CONPs effectively scavenged ROS and promoted M2 polarization by regulating the *CGRP/AMP1/AKT* signaling pathway. MnO_2_ NP is another promising nanozyme for SCI repair. MnO_2_ NP catalyzes the decomposition of H_2_O_2_ into H_2_O and O_2_.^[^ ^131^ ^]^ Li et al.^[^ ^132^ ^]^ incorporated MnO_2_ NP into a peptide‐modified HA hydrogel, which alleviated oxidative stress in the SCI microenvironments and enhanced the survival of transplanted mesenchymal stem cells (MSCs). This approach promoted MSC integration and neural differentiation, leading to significant regeneration of spinal cord tissue. In a related study, hydrogels supplemented with manganese ions also demonstrated antioxidant and neuroprotective effects.^[^ ^133^ ^]^ Other nanozymes, such as selenium nanoparticles^[^ ^134^ ^]^ and iridium nanoparticles,^[^ ^135^ ^]^ have also been explored to mitigate oxidative damage in SCI. However, the potential neurotoxicity of nanozymes is a critical consideration, as concentrations exceeding safe thresholds may impair functional recovery in SCI models.^[^ ^130^ ^]^

**Figure 4:**
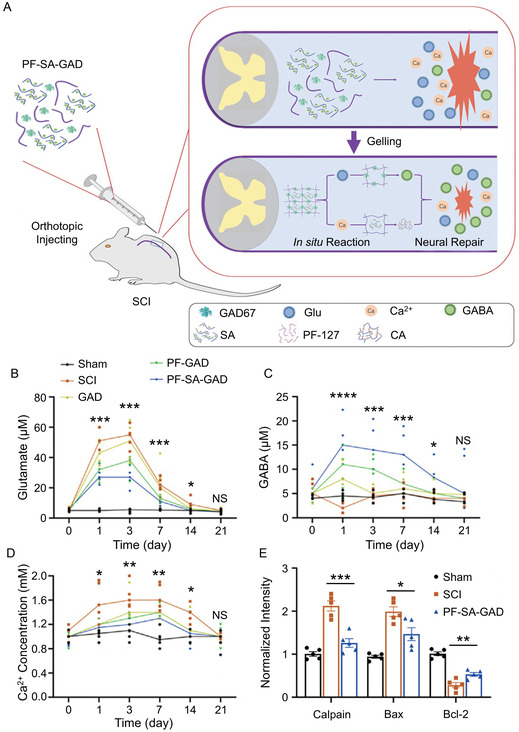
Glu‐ and Ca^2+^‐scavenging hydrogel reduces excitotoxicity and neural death of injured spinal cord. A) In situ assembled trapping gel repairing SCI by capturing glutamate and free Ca^2+^. B) Glu, C) GABA, and D) Ca^2+^ level in CSF of rats. E) Quantification of apoptosis indicated proteins at 28 dpi. All statistical data are represented as mean ± SD (*n* = 5; *P < 0.05, **P < 0.01, ***P < 0.001, ****P < 0.0001). Reproduced with permission.^[^ ^139^ ^]^ Copyright 2023, Wiley‐VCH.

Polymer‐based materials with ROS‐reactive groups also offer ROS‐scavenging capabilities, although their efficiency generally lags behind nanozymes. Nevertheless, these materials can be designed as drug carriers for ROS‐responsive release. The phenylboronic group is a well‐known chemical moiety with ROS‐scavenging properties. Zou et al.^[^ ^13^ ^]^ developed an amphiphilic block copolymer with a hydrophilic poly(ethylene glycol) (PEG) segment and a hydrophobic segment containing phenylboron‐based ROS scavengers designed to encapsulate neural‐targeted prodrugs. These ROS‐scavenging nanoparticles protected spared tissues and axons from secondary injury and remained at the lesion core for nearly 48 h. Notably, nanoparticles injected at 7 dpi exhibited superior prodrug release compared to those injected at 4 or 14 dpi, likely because ROS levels peak around 7 dpi, thereby enhancing the efficiency of drug release. This targeted drug delivery facilitated significant recovery of motor function through localized regulation of spinal circuits. However, the effectiveness of standalone anti‐ROS therapies in restoring motor function is often limited. Combining ROS scavenging with complementary therapeutic mechanisms maximizes functional and histological recovery (**Figure** **4**C−E). The sulfide group is another potent ROS‐scavenging chemical moiety. Zhang et al.^[^ ^136^ ^]^ developed a unique polymer nanosystem (PELPNP) consisting of a phospholipid/phosphorus shell and a sulfur‐containing ether polymer core, synthesized using a thioacetylene click polymerization method. In vitro studies revealed that PELPNP effectively reduced ROS levels and oxidative stress in glial cells and neurons, offering potent anti‐inflammatory and neuroprotective benefits. Upon intravenous administration, PELPNP accumulated at the SCI site via an enhanced permeability and retention‐like effect, efficiently mitigating inflammation and oxidative stress.

### 3.2 Reducing Neural Excitotoxicity

Excitotoxicity in SCI is driven by the excessive accumulation of Glu and the resulting Ca^2+^ imbalance.^[^ ^137^ ^]^ Biomaterials designed to clear Glu/Ca^2+^, repair damaged cell membranes, and enhance mitochondrial function help alleviate this condition.

Dextran, a biomaterial rich in hydroxyl groups, is particularly effective in binding Glu through hydrogen bonding. Under acidic conditions and using *p*‐toluenesulfonate as a catalyst, vicinal diols in dextran can be modified with 2‐methoxypropene, converting water‐soluble dextran into water‐insoluble acetylated dextran (AcDX), making it a promising candidate for Glu clearance. Liu et al.^[^ ^138^ ^]^ utilized a microfluidic flow‐focusing device to produce AcDX microspheres. When intrathecally injected, these microspheres significantly reduced the volume of traumatic lesions and the inflammatory response in injured spinal cords, protected neurons from apoptosis, and restored locomotor function in rats with injuries. In addition to physically adsorbing Glu, the enzymatic conversion of glutamic acid into nontoxic compounds is a promising therapeutic approach. Huang et al.^[^ ^139^ ^]^ developed an in situ‐assembled hydrogel that delivers recombinant glutamate dehydrogenase 67, which converts glutamic acid to GABA. This hydrogel downregulated the proapoptotic proteins Bax and calpain, and upregulated the antiapoptotic proteins Bcl‐2, demonstrating strong neuroprotective effects.

Certain biomaterials chemically SEQUESTer Ca^2+^ through unique reactions. Alginate hydrogels, renowned for their excellent biocompatibility, react with Ca^2+^ to form calcium alginate, allowing for in situ solidification. McKay et al.^[^ ^140^ ^]^ developed a hydrogel combining sodium alginate (SA), chitosan, and genipin. Alginate interacts with extracellular Ca^2+^, triggering gelation among the components and maintaining an elastic modulus (≈1000 Pa) similar to native spinal cord tissue. Huang et al.^[^ ^139^ ^]^ designed a hydrogel using SA and pluronic F‐127 (PF‐127) that interacts with Ca^2+^ at 37 °C, forming a gelable calcium alginate through auto‐exchange of Na^+^ and Ca^2+^ (Figure 4A). This hydrogel (PF‐SA‐GAD) was used to load rGAD67, effectively scavenging Ca^2+^ and Glu. By 3 dpi, PF‐SA‐GAD reduced cerebrospinal fluid Ca^2+^ levels from 1.6 to 1.0 mM and Glu levels from 55 to 25 μM (Figure 4B−E). Additionally, ferulic acid,^[^ ^141^ ^]^ glycol chitosan,^[^ ^141^ ^]^ and C_60_^[^ ^142^ ^]^ have shown neuroprotective effects against excitotoxicity, though their mechanisms remain unclear.

PEG, widely recognized for its membrane‐sealing properties, has been utilized to restore damaged membranes and reduce Ca^2+^ influx, thereby promoting recovery of motor function. However, high concentrations and extremely short half‐lives (<10 min) often limit its application. To address this, Shi et al.^[^ ^143^ ^]^ developed self‐assembled micelles from monomethoxy PEG‐poly(D,L‐lactic acid) di‐block copolymers. These micelles (60 nm in diameter) comprise a hydrophilic PEG shell and a hydrophobic core. Injured spinal tissue treated with these micelles showed a rapid restoration of compound action potentials and a reduced Ca influx, requiring lower concentrations than those of PEG. Notably, compound action potentials continued to improve over 40 min post‐injury.

Mitochondrial dysfunction and ATP depletion following SCI impair Ca^2+^ clearance, contributing to excitotoxicity.^[^ ^144^ ^]^ Zhao et al.^[^ ^145^ ^]^ synthesized polymer quantum dot (Pdot) through a green synthesis approach using polyvinylpyrrolidone precursors and copper catalysis. These Pdot significantly improved mitochondrial function in SCI mice by activating the *AMPK/PGC‐1α/Sirt3* pathway, which restored mitochondrial integrity and reduced Ca overload. This offers a potential avenue for developing antiexcitotoxic therapies.

### 3.3 Restoring Blood−Spinal Cord Barrier

The BSCB exhibits self‐healing properties, beginning to restore around 7 dpi.^[^ ^146^ ^]^ However, the BSCB's opening during this period facilitates the infiltration of inflammatory cells and the entry of toxic molecules, leading to tissue edema and neuronal damage.^[^ ^77^ ^]^ Thus, early BSCB repair is crucial for preserving spinal cord function, and the current strategies for BSCB restoration focus on preventing cell death and promoting vascular regeneration.

**Figure 5:**
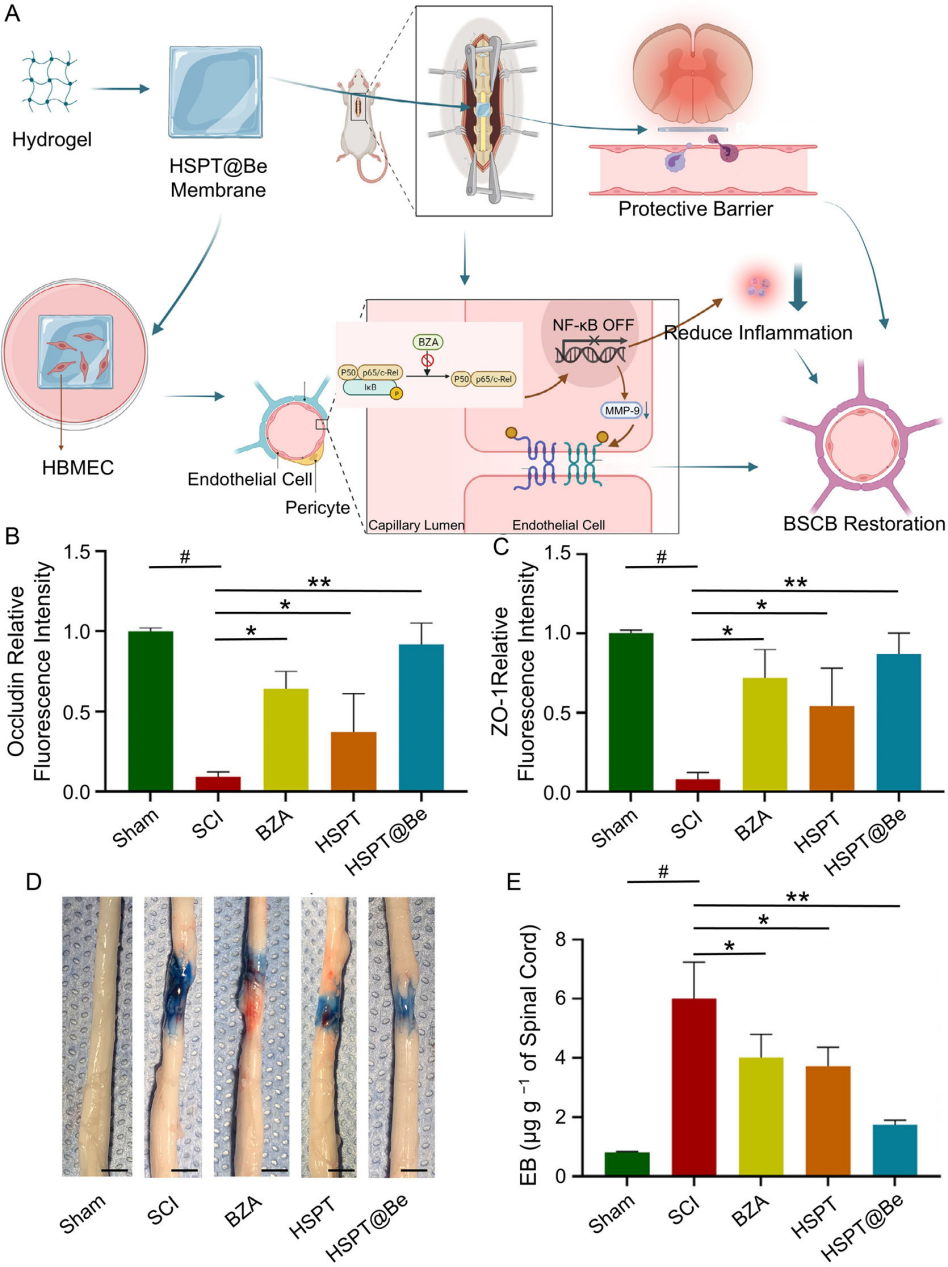
HSPT@Be restores the permeability of BSCB. A) Synthesis of HSPT@Be and mechanism of BSCB repair. B) Occludin and C) ZO‐1 expression in the spinal cord after treatment with BZA, HSPT, and HSPT@Be. D) Representative images of the spinal cord and E) quantification of EB dye extravasation into the spinal cord at 1 dpi (scale bar = 1 mm). All statistical data are represented as mean ± SD (*n* = 5; *P < 0.05, **P < 0.01, ^#^ *P* < 0.05 compared with the sham group). Reproduced with permission.^[^ ^147^ ^]^ Copyright 2023, Elsevier.

Some pharmacological strategies mitigate endothelial cell death nonspecifically by suppressing inflammatory responses. For instance, bazedoxifene (BZA), a third‐generation estrogen receptor modulator, has shown potential for repairing CNS injuries. However, the direct administration of BZA into the subdural or subarachnoid space near the injured spinal cord often results in rapid diffusion due to cerebrospinal fluid flow, which limits its therapeutic effectiveness for SCI. To address this issue, Xin et al.^[^ ^147^ ^]^ developed a novel biomaterial‐based carrier system, constructing a BZA‐loaded HSPT (HA, SA, polyvinyl alcohol (PVA), and tetramethylpropane (TPA) material construction) complex (HSPT@Be), to enhance targeted BZA delivery to SCI sites (**Figure** **5**A). The system was found to inhibit oxidative stress, reduce inflammation, and upregulate TJ proteins, including occludin and ZO‐1 (Figure 5B,C). Evans blue (EB) dye assays confirmed that HSPT@Be significantly restored the damaged BSCB (Figure 5D,E). Similarly, Joshi et al.^[^ ^148^ ^]^ developed carbon monoxide releasing molecule‐2 (CORM‐2)‐loaded solid lipid nanoparticle (LNP) to promote endogenous CO release. This approach reduced immune cell activation, preserved BSCB integrity, and prevented endothelial cell damage. Additionally, certain types of exosomes have shown promise in BSCB repair. For example, exosomes derived from Treg cells deliver miR‐2861 to the injured spinal cord, inducing the expression of vascular TJ proteins through negative regulation of IRAK1 expression.^[^ ^149^ ^]^ Exosomes from bone marrow MSCs protect the BSCB by inhibiting pericyte pyroptosis.^[^ ^150^ ^]^ However, these strategies often lack specificity, limiting their overall efficacy in BSCB repair.

For more efficient BSCB restoration, promoting vascular regeneration is critical. CD146 and CD271, essential during the BSCB development, have become therapeutic targets. Xie et al.^[^ ^151^ ^]^ identified exosomes derived from CD146^+^CD271^+^ human umbilical cord mesenchymal stem cells (hUC‐MSCs) subpopulations as potent agents for the BSCB repair. To enhance their targeting capability, the exosomes were engineered to express the Arg–Gly–Asp peptide, which specifically binds to integrin αv*β*3, expressed on endothelial cells during pathological angiogenesis but not in normal tissues. The engineered exosomes effectively targeted neovascularization in SCI mice, stabilized the BSCB, and improved functional recovery. Mechanistically, the *miR‐501‐5p/MLCK* axis played a key role in this process. Furthermore, Wang et al.^[^ ^152^ ^]^ discovered a novel target: Sox17, predominantly localized in endothelial cells and significantly upregulated after SCI. Using ubiquitin C‐terminal hydrolase L1 to downregulate Sox17, they modulated angiogenesis and promoted BSCB repair.

### 3.4 Reducing Scar Formation

Although scar tissue matures around two weeks post‐injury, scar formation begins as early as 1 dpi, with the mobilization of astrocytes, pericytes, Mφ, and other cell types.^[^ ^26^ ^]^ Early intervention is crucial for minimizing scar formation and removing inhibitory components, thereby facilitating axonal regeneration through the injury site.^[^ ^153^ ^]^ Astrocytes, primary components of glial scars, have been extensively studied in this context. Current methods to reduce glial scar formation involve inhibiting astrocyte activation, preventing EpC differentiation into astrocytes, and employing gene reprogramming to convert astrocytes into neurons.

**Figure 6:**
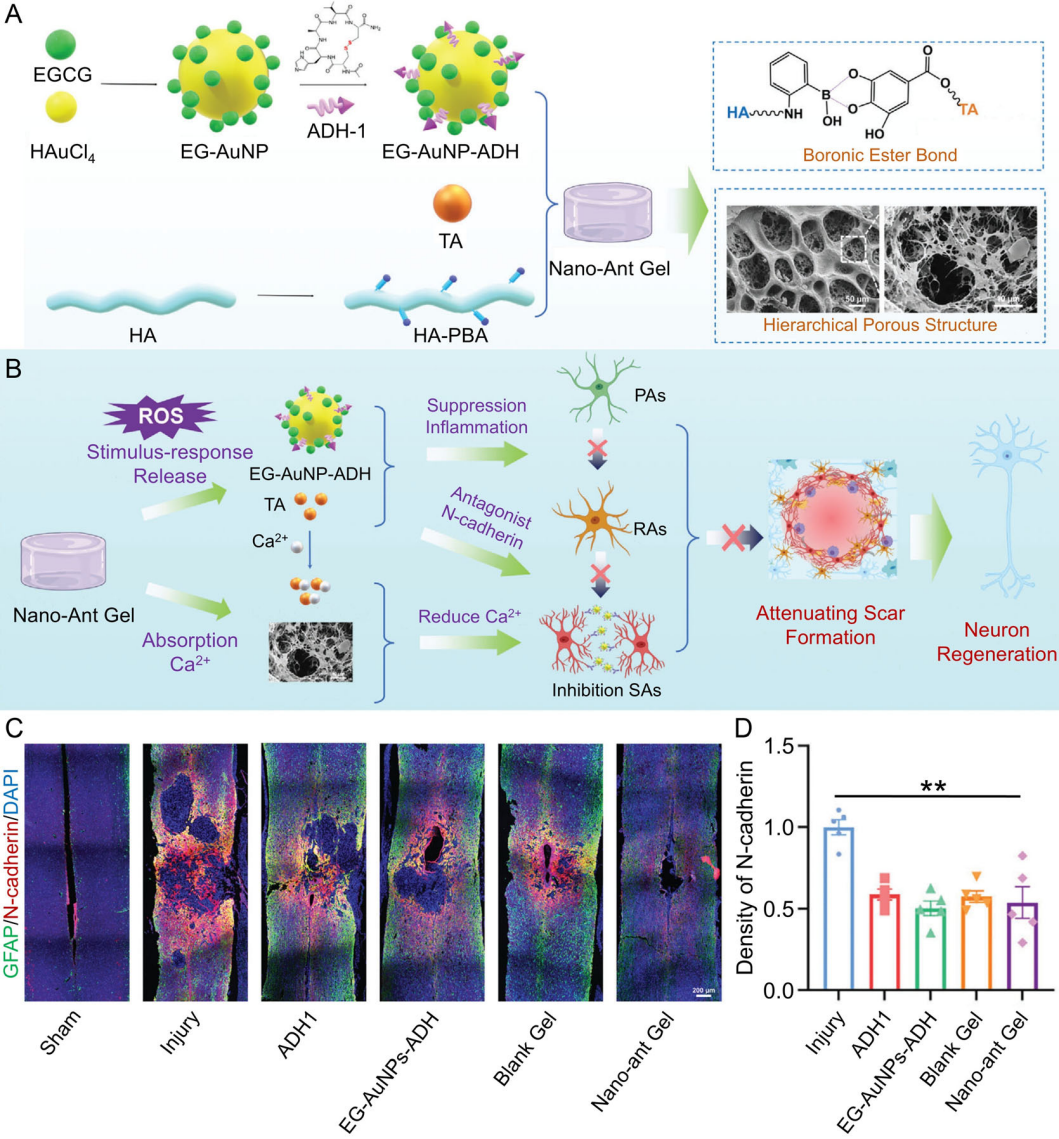
Nano‐ant Gel reduces the glial scar formation by inhibiting inflammation and the antagonist N‐cadherin in the injured spinal cord. A) Synthesis of Nano‐ant Gel. B) Mechanism of Nano‐ant Gel in reducing glial scar formation. C) Images and D) quantification of immunofluorescence staining with GFAP and N‐cadherin. All statistical data are represented as mean ± SD (*n* = 5; **P < 0.01). Reproduced with permission.^[^ ^154^ ^]^ Copyright 2024, Wiley‐VCH.

Inhibiting the inflammatory response and the integrin‐N‐cadherin pathway are effective strategies to suppress astrocyte activation, as these pathways are directly involved in astrocytic phenotypic transitions. For example, a carrier‐free nanomedicine using MS and rutin effectively reduced the expression of active microglia marker Iba‐1 and activated astrocyte marker GFAP at the lesion core.^[^ ^78^ ^]^ CONP reduces inflammatory responses by scavenging ROS and downregulating the expression of GFAP and Mφ biomarker ED1.^[^ ^129^ ^]^ Numerous studies have demonstrated a positive correlation between inflammatory responses and the formation of glial scars.^[^ ^81^, ^91^, ^96^, ^110^, ^136^ ^]^ Recently, Liu et al.^[^ ^154^ ^]^ combined anti‐inflammatory strategies with N‐cadherin signaling inhibition to potently suppress scar formation. Specifically, they loaded the N‐cadherin antagonist ADH‐1 onto (−)‐epigallocatechin‐3‐*O*‐gallate‐gold nanoparticle (EG‐AuNP) to create a nano‐antagonist (EG‐AuNPs‐ADH). This complex was encapsulated into a hydrogel synthesized from HA and tannic acid, resulting in a nano‐antagonist hydrogel (Nano‐ant Gel; **Figure** **6**A). The nano‐ant Gel demonstrated enhanced anti‐inflammatory effects and N‐cadherin antagonism (Figure 6B). Immunofluorescence staining revealed markedly low expression of N‐cadherin and a significantly reduced glial scar area (Figure 6C,D), with the CSPG‐labeled scar area shrinking by nearly 75%.

NSCs, mainly EpCs, have the potential to differentiate into three lineages: astrocytes, neurons, and ODCs. However, under the inhibitory microenvironments of SCI, they primarily differentiate into astrocytes,^[^ ^24^, ^155^ ^]^ which are key contributors to the formation of glial scars. In the early stages of SCI, modulating NSC differentiation to reduce astrocyte production while enhancing neuronal differentiation is crucial for promoting neural repair.^[^ ^24^ ^]^ Therapeutic approaches often involve neurotrophic factors, such as nerve growth factor,^[^ ^156^ ^]^ brain‐derived neurotrophic factor,^[^ ^157^ ^]^ and NT‐3,^[^ ^158^ ^]^ as well as small molecules, including epothilone B^[^ ^159^ ^]^ and paclitaxel (PTX).^[^ ^160^ ^]^ Biomaterial‐based drug delivery systems offer sustained induction signals for NSCs, improving therapeutic outcomes. Furthermore, specific biomaterials themselves inhibit NSC differentiation into astrocytes. For example, a conductive, biocompatible injectable hydrogel composed of agarose, gelatin, and polypyrrole (Aga/Gel/PPy, conductivity: 0.2 S m^−1^) was developed to promote endogenous neurogenesis by activating NSC intracellular Ca^2+^ signaling.^[^ ^161^ ^]^ Additionally, biodegradable germanium phosphide nanosheets were incorporated into an adhesive HA‐graft‐dopamine hydrogel (HA‐DA/Gep@PDA, conductivity: 0.365 S m^−1^). Immunostaining showed enhanced spatial distribution of Tuj‐1‐labeled neurons and reduced GFAP‐labeled astrocytes in the SCI lesion core, with the HA‐DA/Gep@PDA treatment group demonstrating the highest neuronal density and lowest astrocytic density.^[^ ^162^ ^]^

Neuronal reprogramming in vivo has recently emerged as a promising approach to regenerating functional neurons from endogenous glial cells. Puls et al.^[^ ^163^ ^]^ demonstrated successful reprogramming of RAs into neurons in the dorsal horn of SCI models using adeno‐associated virus (AAV)‐mediated *NeuroD1* gene therapy. The conversion efficiency was remarkably high (≈95%). Other genes, such as *Ptbp1* ^[^ ^164^ ^]^ and *Sox2*,^[^ ^165^ ^]^ have shown similar potential for reprogramming glial cells in CNS disorders, although their effectiveness in SCI models remains untested. These targets can be investigated in future studies. Biomaterial‐based regulation of astrocyte‐to‐neuron conversion is still in its infancy, but combining biomaterials with localized delivery systems and precise guidance may enhance the efficiency and clinical applicability of this approach.

Pericytes, similar to astrocytes, are critical contributors to fibrotic scar formation.^[^ ^166^ ^]^ Their secretion of ECM components, such as type IV collagen and laminin,^[^ ^167^ ^]^ significantly hinders axonal regeneration. Genetic manipulation to limit pericyte‐driven fibrotic scarring has shown promise in facilitating axonal growth across lesion sites, though excessive inhibition compromises wound sealing.^[^ ^168^ ^]^ Currently, research on biomaterial‐based modulation of pericytes is limited. However, the development of nanomaterials or hydrogels targeting pericytes presents a promising therapeutic avenue for SCI treatment.

**Figure 7:**
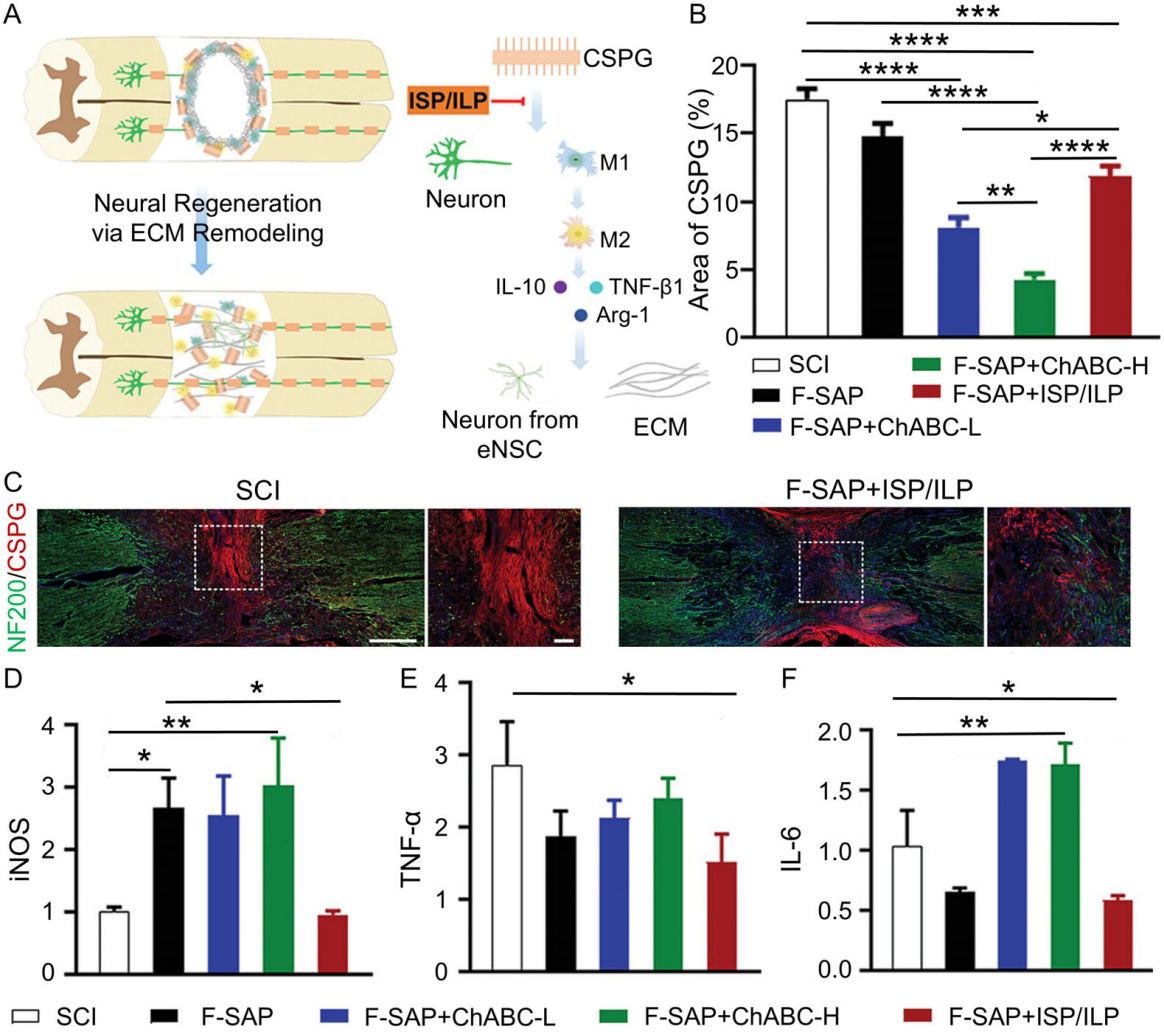
Treatment with blockers of the CSPG receptors promotes neuronal regeneration and induces motor functional recovery of SCI rats. A) Endogenous repair of SCI through local ECM remodeling in lesion core. B) Quantification of CSPG expression in spinal cord (*n* = 6). C) Immunofluorescence images of NF200, CSPG, and DAPI in spinal cord (scale bar = 500 μm and 50 μm). D) iNOS, E) TNF‐α, and F) IL‐6 mRNA expression levels in different groups determined by RT‐PCR. All statistical data are represented as mean ± SD (*n* = 6; *P < 0.05, **P < 0.01, and ***P < 0.001). Reproduced with permission.^[^ ^173^ ^]^ Copyright 2023, Wiley‐VCH.

In addition to targeting cellular components, directly modulating the matrix components of scar tissue has proven to be a more effective strategy. CSPGs are the most critical component of glial scar. Traditionally, axons were believed to stop extending or sprouting once they encountered CSPG‐rich scar boundaries. Since 2002, researchers have extensively attempted to deplete CSPG.^[^ ^169^, ^170^, ^171^, ^172^ ^]^ Although these studies nearly eliminated glial scars, they also caused significant issues, such as the spread of inflammation. As a result, the global CSPG depletion is not considered advisable. Recent studies suggest that blocking CSPG receptors on neuronal membranes, such as PTPσ and LAR, may mitigate these adverse effects.^[^ ^70^ ^]^ Sun et al.^[^ ^173^ ^]^ compared two therapeutic strategies (**Figure** **7**A): using a functional self‐assembling peptide (F‐SAP) hydrogel loaded with membrane‐permeable intracellular sigma peptide (ISP) and intracellular LAR peptide (ILP) to target CSPG signaling inhibition (F‐SAP + ISP/ILP) versus a hydrogel loaded with chondroitinase ABC (F‐SAP + ChABC‐H). In the F‐SAP + ChABC‐H group, CSPG was extensively depleted, while the F‐SAP+ISP/ILP group showed only a modest reduction (Figure 7B). Despite this, the number of NF200^+^ axons in the F‐SAP + ISP/ILP group was nearly 2.5 times higher than in the F‐SAP + ChABC‐H group (Figure 7C). Moreover, Iba‐1^+^ cells in the F‐SAP + ChABC‐H group exhibited significant spread at the epicenter and elevated expression of pro‐inflammatory factors, such as iNOS, TNF‐α, and IL‐6 (Figure 7D−F). In contrast, the F‐SAP + ISP/ILP group had limited Iba‐1^+^ cell distribution and increased expression of anti‐inflammatory factors, including ARG1, TGF‐*β*, and IL‐10. These improved lesion microenvironments fostered endogenous neurogenesis and matrix remodeling, resulting in enhanced motor function in rats with SCI. A similar example is provided by Kong et al.,^[^ ^174^ ^]^ who developed a Janus‐structured layered nanoplatform for dual drug delivery. The platform simultaneously released enoxaparin, which inactivated PTPσ on neuronal membranes and PTX, which stabilized microtubules and promoted axonal growth. This multifaceted approach significantly enhanced axonal regeneration at the lesion site. The rats’ BBB score increased to 15 in the treatment group, compared with 4 in the control group, after 28 days, reflecting a notable improvement in motor function.

Finally, we summarized the mechanism of biomaterials for acute SCI repair in **Table** **2**, including their biological effects, key signaling pathways, and protein interactions.^[^ ^90^, ^95^, ^119^, ^175^, ^176^, ^177^, ^178^, ^179^, ^180^, ^181^, ^182^ ^]^

**Table 2:** Physiological effects and related signaling pathway/key protein interaction of biomaterials for acute SCI repair.

Biomaterial	Physiological effect	Signaling pathway/ key protein interaction	Reference
Ce‐enhanced carbon dot	Mitigating mitochondrial damage; reducing neuronal apoptosis; promoting Mφ polarization from M1 to M2; restoring homeostasis of biometal ions.	Activating *AMPK/PGC‐1α/Nrf‐2* signaling pathway.	[182]
PLGA‐PLLA nanofiber	Reducing M1 polarization of microglia; promoting differentiation of NSCs.	Activating *cGMP–PKG* and *cAMP* signaling pathways.	[181]
Peptide nanofiber (PNF)/chitosan (CS)/VD11 (VDELWPPWLPC) hydrogel	Reducing Mφ activation and astrocyte proliferation; promoting neovascularization; promoting neurogenesis.	Activating *PI3K/AKT/mTOR* signaling pathway.	[180]
Ultra‐small‐diameter lentinan Se NP	Reducing oxidative stress‐induced cytotoxicity; alleviating mitochondrial dysfunction; reducing apoptosis.	Activating *PI3K‐AKT‐mTOR* and *Ras‐Raf‐MEK‐ERK* signaling pathways by regulating selenoproteins.	[179]
LET/TMP/Rg1@Se NP	Reducing oxidative stress‐induced cytotoxicity; reducing inflammatory storm induced by microglial cells.	Inhibiting *NLRP3/caspase‐1* pathway.	[178]
3D injectable electrospun short fiber	Suppressing reactive astrogliosis; inhibiting neuron apoptosis.	Modulating “ECM receptor interaction” pathway, inhibiting transcription of downstream Vimentin protein; blocking binding of Vimentin protein with inflammation‐related proteins, downregulating the *NF‐κB* signaling pathway.	[175]
Ejiao carbon dot	Diminishing local infiltration of monocytes and Mφ.	Downregulating CCAAT enhancer binding protein‐β expression in spleen and upregulating FZD4 protein expression.	[177]
PEG‐CeMn nanozyme	Promoting M2 polarization.	Inhibiting *cGAS‐STING* signaling pathway.	[176]
Cr/Ce@PDA NP	Reprogrameing inflammatory Mφ to proregenerative phenotype; promoting regeneration and differentiation of neural cells.	Inhibiting *succinate/HIF‐1**α**/IL‐1**β*** signaling axis; activating *mTOR* pathway	[119]
Mg/Fe layered double hydroxide NP	Inhibits inflammation; promoting neurogenesis	Downregulating *Peizo1/ NF‐**κ**B* signaling pathway	[95]
Mg/Al‐layered double hydroxide nanoparticle	Promoting M2 polarization; promoting neurogenesis.	Targeting TGFBR2; inhibiting *Smad 2/3* and *MEK/ERK* signaling pathway.	[90]

## 4 Clinical Trials for Acute Spinal Cord Injury

Previously, we discussed animal studies highlighting biomaterial‐based emergency interventions for SCI. In this section, we summarize the current achievements of early treatment methods in clinical trials to inform both basic research and clinical applications.

Over the past two decades, significant advances have been made in SCI recovery using experimental models, but few therapies have been validated for human use.^[^ ^183^ ^]^ MS is the only US food and drug administration (FDA)‐approved drug specifically for acute SCI treatment.^[^ ^184^ ^]^ However, the latest AO guidelines recommend MS infusion only within 8 h of injury,^[^ ^185^ ^]^ highlighting the limited clinical availability of emergency interventions for SCI. Ongoing clinical trials for early pharmacological interventions include glyburide (NCT02524379), CRIS100 (NCT05739734), TZ‐161 (NCT06677229), sovateltide (NCT04054414), MT‐3921 (NCT04096950), elezanumab (NCT04295538), TNFα monoclonal antibody (NCT04988425), and ALMB‐0166 (NCT05524103; **Table** **3**). These trials primarily target early‐stage neuroinflammation and neurotoxicity, but further experimental validation is needed to determine their efficacy in promoting SCI recovery.

**Table 3:** Clinical trials of acute SCI treatment.

Drug/Biomateriala)	Combination therapy	Injury location	AISA	Phase	Status	Year	NCT number
Glyburide	None	C2‐C8 SCI	A‐C	I/II	Terminated	2017	NCT02524379
Sovateltide	None	C5‐S5 SCI	B‐D	II	Recruiting	2019	NCT04054414
MT‐3921	None	C4‐8 SCI	A‐C	I	Completed	2019	NCT04096950
Elezanumab	None	C4‐7 SCI	A‐B	II	Active, not recruiting	2020	NCT04295538
MT‐3921	None	C4‐7 SCI	A‐C	II	Active, not recruiting	2021	NCT04683848
TNF‐α monoclonal antibody	None	SCI	A‐D	I/II	Unknown status	2022	NCT04988425
Glyburide	None	C2‐T12 SCI	A‐C	I	Recruiting	2022	NCT05426681
ALMB‐0166	None	C4‐T12 SCI	B‐C	I	Unknown status	2022	NCT05524103
CRIS100	None	T2‐T10 SCI	A	Early I	Not yet recruiting	2023	NCT05739734
TZ‐161	None	T1‐T12 SCI	B‐D	I/II	Not yet recruiting	2024	NCT06677229
Neurospinal scaffold (PLGA‐PLL)	None	T2‐T12 SCI	A	Not applicable	Terminated	2014	NCT02138110
Functional neural regeneration collagen scaffold	hUC‐MSCs/BMMCs	C4‐T12 SCI	A	I	Unknown	2015	NCT02510365
Neurospinal scaffold (PLGA‐PLL)	None	T2‐T12 SCI	A	Not applicable	Terminated	2019	NCT03762655
Functional neural regeneration collagen scaffold	Epidural electrical stimulation	C2‐L1 SCI	A	I/II	Unknown	2019	NCT03966794

a) All clinical trials in the table can be found on the https://www.clinicaltrials.gov/.

Biomaterials have demonstrated promising results in experimental studies, though most have been tested only in rodent models. Transitioning these materials for safe and effective human use remains a significant challenge. Current clinical trials investigating biomaterial applications for acute SCI include NCT02138110, NCT02510365, NCT03762655, and NCT03966794 (Table 2). All involve implantable scaffolds, with no trials involving nanoparticles to date.

InVivo Therapeutics Corp (Cambridge, Massachusetts) developed a bioresorbable polymer scaffold, known as the Neuro‐Spinal Scaffold, composed of PLGA covalently bonded to poly(L‐lysine) (PLL). The biocompatibility and efficacy of the scaffold were first validated in rat models, demonstrating sustained functional recovery lasting up to one year.^[^ ^186^ ^]^ Subsequent validation using a large animal model (African green monkeys) revealed no significant adverse effects for over 80 days post‐implantation, accompanied by the restoration of hindlimb motor function.^[^ ^187^ ^]^ This scaffold was the first reported polymer scaffold‐implanted in the SCI site of patients in 2014 (NCT02138110). Its primary function is to minimize necrotic expansion and provide structural support for surviving tissue.^[^ ^188^ ^]^ Through 24 months of follow‐up, the acute implantation of the Neuro‐Spinal Scaffold into injured spinal cords demonstrated a favorable safety profile. Improvements in the AIS grade were observed in seven of 16 patients (43.8%).^[^ ^189^, ^190^ ^]^ The study also demonstrated that scaffold implantation in humans is safe over 24 months. To further assess the scaffold's potential benefits for safety and neurological recovery in patients with thoracic complete SCI compared to standard‐of‐care spine surgery, a new clinical trial involving 20 patients was initiated in 2019 (NCT03762655). At the 6‐month follow‐up, no adverse effects were observed. Regarding neurological improvement at six months post‐surgery, AIS grade improvement was seen in two scaffold‐implanted patients (20%; both AIS C) and three control group patients (30%; AIS B [*n* = 2] or AIS C [*n* = 1]). The results show that the bioresorbable scaffold did not demonstrate a clear clinical benefit. Consequently, this clinical trial was discontinued.

Dai's group developed a linearly ordered collagen scaffold, the NeuroRegen Scaffold, designed to replace the injured microenvironments and serve as a carrier for transplanted cells in the injured spinal cord.^[^ ^191^ ^]^ They conducted extensive preclinical testing in rodent,^[^ ^192^, ^193^ ^]^ canine,^[^ ^194^, ^195^ ^]^ and nonhuman primate^[^ ^196^ ^]^ models to evaluate the scaffold's safety and efficacy. Based on these results, a clinical trial (NCT02510365) was initiated in 2015 for patients with acute SCI. When loaded with hUC‐MSCs and implanted in two patients with acute SCI, the NeuroRegen Scaffold yielded significant improvements in sensory and motor functions after one year of follow‐up, with AIS grades improving from A to C.^[^ ^197^ ^]^ Similarly, the scaffold, when loaded with autologous bone marrow mononuclear cells (BMMCs) and implanted in seven patients with thoracic SCI, improved sensation and autonomic functions. However, motor function remained unchanged after three years.^[^ ^198^ ^]^ The NeuroRegen Scaffold demonstrated no long‐term adverse effects. Furthermore, they used the NeuroRegen Scaffold combined with epidural electrical stimulation to treat acute and chronic SCI patients in 2019 (NCT03966794). Despite these promising results, overall locomotor recovery in patients remains limited.

## 5 Summary and Perspective

Emergency intervention for SCI aims to limit secondary damage and preserve viable cells to support neurological recovery. Preserving functional components is essential for restoring neural function. Biomaterials have been extensively applied to address early pathological events in SCI by inhibiting immune responses, reducing excitotoxicity, restoring the BSCB, and minimizing scar formation. Over the past two decades, significant progress has been made. However, given the complex pathology of SCI, reliable methods to fully restore lost neural function remain scarce. Future biomaterial‐based interventions must overcome several key challenges.

First, a limited understanding of effective therapeutic targets hampers biomaterial efficacy. Identifying effective treatment strategies requires deeper foundational research. Species differences may provide important insights. For example, zebrafish exhibits robust regenerative capabilities, quickly recovering swimming ability after SCI.^[^ ^199^ ^]^ Mechanistically, within the first 12 hpi, inflammation and IL‐1*β* promote axonal bridging. Between 12 and 48 hpi, IL‐1*β* levels decrease, while anti‐inflammatory cytokines TGF‐*β*1a and TGF‐*β*3 increase, thereby suppressing inflammation. This biphasic immune response, regulated by peripheral Mφ, suggests that IL‐1*β* and Mφ could be promising therapeutic targets.^[^ ^199^ ^]^ Age‐related differences also provide critical clues. Young individuals demonstrate stronger neural recovery compared to adults. For example, Li et al.^[^ ^200^ ^]^ reported that neonatal mice exhibit scar‐free healing, allowing axonal growth through the lesion site. This regeneration is associated with the secretion of fibronectin and the upregulation of specific peptidase inhibitors by microglia. Additionally, successful therapeutic approaches from other CNS conditions, such as brain and optic nerve injuries, may inform SCI interventions.

Second, the lack of suitable research models limits both basic studies and the validation of treatment efficacy. Most SCI research relies on rodent models, which differ significantly from humans in age, weight, genetics, and physiology. These differences present significant obstacles to clinical translation. To bridge this gap, research using large animal models, particularly nonhuman primates that closely mimic human biology, is crucial for advancing theoretical research and preclinical evaluation of biomaterials. Organoids, as an advanced in vitro technology, replicate key human tissue characteristics, including three‐dimensional (3D) structure, intercellular connections, and genetic fidelity. The development of spinal cord organoids (SPOs) offers a promising model for SCI research, closely mirroring human biological features.^[^ ^201^, ^202^, ^203^, ^204^ ^]^ Human SPOs provide valuable insights into the pathological mechanisms of human SCI and serve as effective platforms for testing therapeutic interventions, significantly enhancing clinical translation potential. Moreover, clinical samples, such as cerebrospinal fluid and surgically excised intervertebral discs, represent valuable resources for developing future therapeutic strategies.^[^ ^42^ ^]^ Another notable limitation of current preclinical and clinical trials is an emphasis on middle and lower thoracic SCI, whereas cervical SCI is more prevalent in clinical settings. To better address patient needs, future research should prioritize disease models that more accurately reflect real‐world clinical conditions.

Third, a major obstacle to advancing SCI treatments lies in ineffective clinical delivery methods. The design of therapeutic biomaterials must consider their translational potential. Intravenous nanomedicine, a noninvasive approach with high patient acceptance, is promising but requires minimal systemic toxicity. Additionally, most SCI patients undergo early spinal canal decompression surgery, presenting an opportunity for noninvasive or minimally invasive interventions, such as biomaterial‐based patches, microneedles, or injectable hydrogels, which minimize patient discomfort. Combination medicines may be utilized in future strategies, with biomaterials implanted directly at the injury site during surgery to optimize the microenvironments and intravenous nanomedicine administered post‐operatively for precise dosing and timing, thereby facilitating personalized treatment regimens. Emerging insights into novel drug delivery mechanisms further expand therapeutic options. The gut microbiota, CNS communication, mediated by the microbiota‐gut‐brain axis,^[^ ^205^ ^]^ plays a role in various CNS disorders, including stroke,^[^ ^206^ ^]^ multiple sclerosis,^[^ ^207^ ^]^ and Alzheimer's disease.^[^ ^208^ ^]^ Recent studies highlighted the therapeutic potential of gut microbiota‐derived metabolites. For instance, the gram‐positive gut bacterium *Clostridium sporogenes* and its metabolite, indole‐3‐propionic acid (IPA), have shown immune‐mediated benefits following sciatic nerve injury in mice. Oral IPA administration enhances metabolic activity, promoting axonal regeneration and sensory recovery.^[^ ^209^ ^]^ This discovery underscores the potential of oral drug delivery, and further research into the relationship between gut microbiota and SCI may reveal novel therapeutic targets.

In addition, a single treatment approach is often insufficient for meaningful recovery in SCI patients, making combination therapies an inevitable trend. Neuromodulation technologies, such as functional electrical stimulation and brain–machine interfaces, are rapidly evolving and have demonstrated significant improvements in functional recovery, including upper limb movement, hand dexterity, and bladder control.^[^ ^210^, ^211^, ^212^ ^]^ Similarly, stem cell transplantation plays a dual role by modulating immune responses and differentiating into neurons to facilitate the reconstruction of neural circuits.^[^ ^213^ ^]^ Combining biomaterials with neuromodulation and stem cell transplantation has demonstrated enhanced therapeutic outcomes due to their ability to remodel the injured microenvironments.

Furthermore, the risk associated with the clinical application of biomaterials is a key concern. Potential biological toxicity is one of the main obstacles to clinical application. The immunogenicity of biomaterials is a common issue, and cationic polymers (e.g., PEI and PAMAM)^[^ ^19^, ^20^ ^]^ and metal‐based nanozymes have been shown to exhibit specific biological toxicity.^[^ ^130^ ^]^ By strictly controlling the dosage of biomaterials and performing surface modifications (e.g., PEGylation), such problems may be greatly avoided. Prioritizing the use of natural materials also effectively avoids severe immune reactions. Additionally, improper degradation of the scaffold/hydrogel is another potential risk.^[^ ^83^ ^]^ The initially filled spinal cord tissue loses its support due to the degradation of the scaffold/hydrogel, which may cause damage to the regenerated tissue or even the original healthy tissue. Therefore, strict control of degradation is crucial and must be repeatedly verified in preclinical experiments.

Lastly, fully leveraging recent cutting‐edge technologies is expected to enhance the development of biomaterial‐based SCI treatment strategies significantly. Artificial intelligence (AI), as one of the most prominent emerging technologies, offers substantial potential. First, biomaterial design involves numerous complex parameters, such as the topology and mechanical properties of hydrogels and scaffolds, as well as the particle size, surface charge, surface modification, and crystallinity of nanoparticles. These parameters critically influence the biological behavior of neurons, NSCs, and immune cells. Machine learning (ML) leverages vast existing experimental datasets to predict outcomes. For instance, Zhu et al.^[^ ^214^ ^]^ developed a system combining high‐throughput flow‐based single‐cell imaging with deep learning to predict NSC differentiation fates. This system utilizes only bright‐field single‐cell images to forecast the outcomes of NSC differentiation stimulated by common neurotrophic factors, drugs, or nanoparticles. Future efforts could utilize ML models to predict the therapeutic efficacy of biomaterials in SCI, enabling the rational design of highly effective treatment strategies by identifying optimal parameter sets. Furthermore, ML analysis incorporates biomaterial safety profiles and production costs, facilitating clinical translation. 3D printing technology represents another promising approach. The effectiveness of 3D‐printed implantable scaffolds has been increasingly validated.^[^ ^215^, ^216^, ^217^ ^]^ Personalized scaffold design is likely to be a key future trend. First, scaffolds can be designed to precisely match the geometry of lesion cavity in individual patients, particularly when combined with scar tissue removal, thereby significantly enhancing implantability.^[^ ^218^ ^]^ Furthermore, scaffolds can be combined with customized combinations of drugs, growth factors, or even autologous cells, optimizing both structural and biochemical support to promote neural regeneration tailored to the specific injury in individual patients.

In conclusion, biomaterial‐based emergency interventions face significant translational challenges, as only two types of biomaterials have undergone clinical trials to date. However, they are still promising avenues. Individualized treatment for patients is the future direction, particularly the combination of biomaterials with emerging technologies, such as organoids, AI, and 3D printing. By integrating the unique molecular features, cell behaviors, and lesion characteristics of patients, personalized biomaterial design can be achieved to maximize the benefits of treatment for each patient. To achieve these goals, interdisciplinary collaboration among clinicians, rehabilitation specialists, materials scientists, neuroscientists, and engineers will be critical to driving progress in SCI treatment and care in the future.

## Conflict of Interest

The authors declare no conflict of interest.

## Author Contributions

**Jincheng Li:** conceptualization, writing—original draft, writing—review & editing. **Qingzheng Zhang:** writing—review & editing. **Zongtai Liu:** writing—review & editing. **Weiguo Xu:** writing—review & editing. **Changfeng Fu:** conceptualization, writing—review & editing, supervision, project administration, funding acquisition. **Jianxun Ding:** conceptualization, writing—review & editing, supervision, project administration, funding acquisition.
